# Identification of Differentially Expressed Serum Proteins in Infectious Purpura Fulminans

**DOI:** 10.1155/2014/698383

**Published:** 2014-02-10

**Authors:** Ting He, Jiong-yu Hu, Jian Han, Dong-xia Zhang, Xu-pin Jiang, Bing Chen, Yue-sheng Huang

**Affiliations:** ^1^Institute of Burn Research, Southwest Hospital, Third Military Medical University, State Key Laboratory of Trauma, Burns and Combined Injury, Chongqing Key Laboratory for Diseases Proteomics, Chongqing 400038, China; ^2^Department of Endocrinology, Southwest Hospital, Third Military Medical University, Chongqing 400038, China; ^3^Department of Obstetrics and Gynecology, Research Institute of Surgery, Daping Hospital, Third Military Medical University, Chongqing 400042, China

## Abstract

Purpura fulminans (PF) is a life-threatening hemorrhagic condition. Because of the rarity and randomness of the disease, no improvement in treatment has been made for a long time. In this study, we assessed the serum proteome response to PF by comparing serum proteins between healthy controls and PF patient. Liquid chromatography with tandem mass spectrometry (LC-MS/MS) approach was used after depleting 6 abundant proteins of serum. In total, 262 proteins were confidently identified with 2 unique peptides, and 38 proteins were identified significantly up- (≥2) or downregulated (≤0.5) based on spectral counting ratios (SpC_PF/N_). In the 38 proteins with significant abundance changes, 11 proteins were previously known to be associated with burn or sepsis response, but 27 potentially novel proteins may be specifically associated with PF process. Two differentially expressed proteins, alpha-1-antitrypsin (SERPINA1) and alpha-2 antiplasmin (SERPINF2), were validated by Western blot. This is the first study where PF patient and healthy controls are compared in a proteomic study to elucidate proteins involved in the response to PF. This study provides an initial basis for future studies of PF, and the differentially expressed proteins might provide new therapeutic targets to decrease the mortality of PF.

## 1. Introduction

Purpura fulminans (PF) is a rapidly progressive thrombotic disorder with a high mortality. Clinical presentation shows acute disseminated intravascular coagulation (DIC), hemorrhagic necrosis of the skin, hypotension, and fever [1, 2]. PF may herald multiple organ failure (MOF) caused by thrombotic occlusion of small- and medium-sized blood vessels. In children, PF is usually caused by a hereditary deficiency of protein C, protein S, and antithrombin III involved in the coagulopathy pathogenesis. In adults, this syndrome is uncommon [3, 4] and is often associated with a severe acute infection, especially meningococcus, staphylococcus aureus, and streptococcus infections [5–7]. However, the infection alone does not completely explain PF development or the rapidly progressive thrombotic disorder. Hence, characterization of proteins with new and undefined roles in PF pathophysiology is critical for more accurate diagnosis and/or treatment of this disease.

High throughput proteomics approaches raise the possibility of studying complex human diseases and elucidating the underlying mechanisms, helping to discover novel proteins as predictors or new therapeutic targets [8]. In clinical research, serum proteomics is a promising field because blood serum is easily and noninvasively accessible and is a reservoir for circulating proteins throughout the body [9]. The serum proteomics analysis can reveal protein alterations caused by specific pathological conditions [10], especially in blood diseases.

Our department came across an adult woman who developed deadly PF following alcohol flame burn [11]. The erythematous macules rapidly evolved to blue-black haemorrhagic necrosis and she died from MOF on the sixth day after diagnosis. Considering the rarity of the disease, the blood sample collected on the fourth day after diagnosis was kept after obtaining the consent of the immediate family.

In order to clarify the complex pathophysiological mechanisms, we performed a proteomics comparison between the PF patient and healthy individuals using a label-free HPLC-Chip/MS approach. We then applied bioinformatics technologies to analyze the biological responses, cellular components, molecular functions, and potential signal pathways involved in PF. Two proteins participating in coagulation regulation were validated by Western blot. This study may provide an initial basis to understand PF pathogenesis and therefore hopefully yield novel therapeutic targets.

## 2. Materials and Methods

### 2.1. Human Serum Sample Preparation

All serum samples were provided by the Burn Department of the Southwest Hospital. The samples for verification corresponded to healthy control individuals (*n* = 3, females), PF patient secondary to burn sepsis (*n* = 1, female), and burn sepsis patients (*n* = 3, females). All the individuals were Han Chinese and the healthy individuals were clinically evaluated to address normal coagulation function and routine blood tests. A brief summary of patient demographics was provided in Table 1. Blood samples (5 mL) used for proteomics studies were collected by venipuncture and clotted for 30 min followed by centrifugation at 3,000 g for 15 min. Serum was then freshly frozen at −80°C until use. All individuals gave written informed consent, whose creation was performed in accordance with the Helsinki Declaration and approved by the Ethics Committee of the Southwest Hospital, Third Military Medical University.

As described [12], serum aliquots (10 *μ*L) of each control sample were pooled to emphasize proteomic similarities while diluting potential individual contributions [13]. The pooled sample and the PF patient sample (30 *μ*L) were centrifuged at 10,000 ×g for 30 min at 4°C to remove any cellular debris. The targeted high abundance proteins, including albumin, IgG, antitrypsin, IgA, transferrin, and haptoglobin in the samples were depleted by using an immunoaffinity column (Agilent Technologies, USA). The protein concentrations were determined by the Coomassie Protein Assay Kit (Pierce, USA).

### 2.2. LC ESI-MS/MS Analysis

A total of 200 *μ*g serum protein mixtures were digested in solution as described above. After being desalted by reverse phase C18 StageTips, the protein digest (10 *μ*L) was injected to a strong cation exchange (SCX) column (300 *μ*m internal diameter × 5 cm long; Agilent Technologies, Germany) and eluted with 10 salt plug injections (5–500 mM NaCl) [14]. Eleven fractions obtained from 10 salt plug injections were then introduced into the HPLC-Chip/XCT Ultra Trap (Agilent Technologies, USA). The first four fractions were separated with two-hour-long gradients (2% B to 40% B in 110 min and 40% B to 95% B in 10 min, where solvent B is 10% water with 0.1% formic acid, 90% acetonitrile) and the remaining fractions with one-hour-long gradient.

### 2.3. HPLC-Chip/MS Analysis

The tryptic digested and desalted protein samples were analyzed with an HPLC-CHIP-MS/MS system (Agilent Technologies, USA). Peptides were injected on the enrichment column via an autosampler [15]. The mobile phase consisted of solvents A (water with 0.1% formic acid) and B. The column was eluted with a gradient from 3% B to 45% B in 90 min, followed by a steep gradient to 80% B in 10 min. The total analysis time was 110 min, and the flow rate was fixed at 0.3 *μ*L/min. (The MS parameters could be found in the Supplementary Materials available online at http://dx.doi.org/10.1155/2014/698383.)

### 2.4. Database Search

Database searches were performed against the IPI human database (http://www.ebi.ac.uk/IPI) with the Spectrum Mill Proteomics Workbench Rev A.03.03.078 software (Agilent Technologies, USA). All protein hits found in a distinct database search by Spectrum Mill are nonredundant. To eliminate redundancy, the Protein Summary Mode groups all proteins that have at least one common peptide, and only the highest scoring member of each protein group is shown and counted in the protein list. (The database search parameters setting could be found in the supplementary materials.)

### 2.5. Protein Expression Analysis

Spectral counting (SpC) was used to estimate protein abundance and to compare the expression of the same protein among different samples [16]. Normalized SpC (nSpC) values were obtained by dividing the spectral counting of a given protein by the total number of SpCs detected for a particular sample group. Then, the SpC_PF/N_ was calculated according to the following formula: SpC_PF/N_ = nSpC_PF_/nSpC_N_. To avoid dividing by zero errors in the calculations, zero spectral count values were replaced with an empirically determined fractional value of 0.16 [17]. Proteins were considered to have significant expression changes if the SpC_PF/N_ ≥ 2 (overexpressed in PF patient) or SpC_PF/N_ ≤ 0.5 (underexpressed in PF patient) [18].

Bioinformatics strategy was used to integrate the data. Functional classification of differentially expressed proteins was performed using the DAVID software (http://david.abcc.ncifcrf.gov/). DAVID, a high-throughput and integrated data-mining environment, could be used to analyze gene lists derived from high-throughput genomic experiments [19]. The functional classification includes Gene Ontology (GO, including biological processes, cellular components, and molecular functions) analysis and physiological pathways enrichment analysis based on the KEGG (http://www.genome.jp/kegg/) and BioCarta (http://www.biocarta.com/) databases.

### 2.6. Validation by Western Blot

Western blot analysis was performed to ensure the reliability of the HPLC-chip/MS results. The protein concentrations were determined by RCDC protein assay (Bio-Rad, USA). 30 *μ*g or 10 *μ*L serum was separated by 8% SDS-PAGE and transferred onto polyvinylidene difluoride (PVDF) membranes (Millipore, USA). Rabbit anti-alpha-1-antitrypsin (SERPINA1, Proteintech Group Inc., USA) and anti-alpha-2-antiplasmin (SERPINF2, Proteintech Group Inc., USA) were primary antibodies for the immunodetection. HRP-conjugated goat-anti-rabbit was used as secondary antibody (Sigma Aldrich, USA). Bands were visualized by chemiluminescence with ChemiDoc XRC^+^ Imaging System (Bio-Rad, USA) using ECL detection reagents (GE Healthcare, USA). Quantification of the detected bands density value was performed using the Quantity One software (version 4.6.2, Bio-Rad, USA). All immunoblots were run at least in triplicate.

### 2.7. Statistical Analysis

The Fisher exact test was used to perform the enrichment analysis, and the two-tailed one-way analysis of variance (ANOVA) post hoc tests were applied in the Western blot verification. In all comparisons, *P* < 0.05 was considered statistically significant. Statistical analyses were performed with SPSS 19.0 (SPSS Inc., USA) and PASS 11 (NCSS, USA).

## 3. Results

### 3.1. Proteomics Analysis by HPLC-Chip/MS

In order to get valuable pathophysiological information about PF, serum samples from a PF patient and healthy individuals were analyzed by HPLC-Chip/MS and inferred by the label-free methodology of SpC [20–22]. The identified proteins in the healthy controls were used for comparisons. In total, 262 proteins were confidently identified in healthy individuals and the PF patient serum samples with at least two unique peptides (PDF S1). Of those, 38 proteins were differentially expressed between groups with SpC_PF/N_ ≥ 2 or SpC_PF/N_ ≤ 0.5. Among these proteins, 27 proteins have not been reported with clear implications in burn injury or sepsis [23–25]. These potentially PF-associated proteins are listed in Table 2.

### 3.2. Enrichment Analysis of Differentially Expressed Proteins by DAVID

The obtained differentially expressed proteins were converted to the official gene symbol of Entrez Gene and then submitted to DAVID for GO analysis, including biological processes, cellular components, and molecular functions and physiological pathways enrichment analysis.

**Figure 1:**
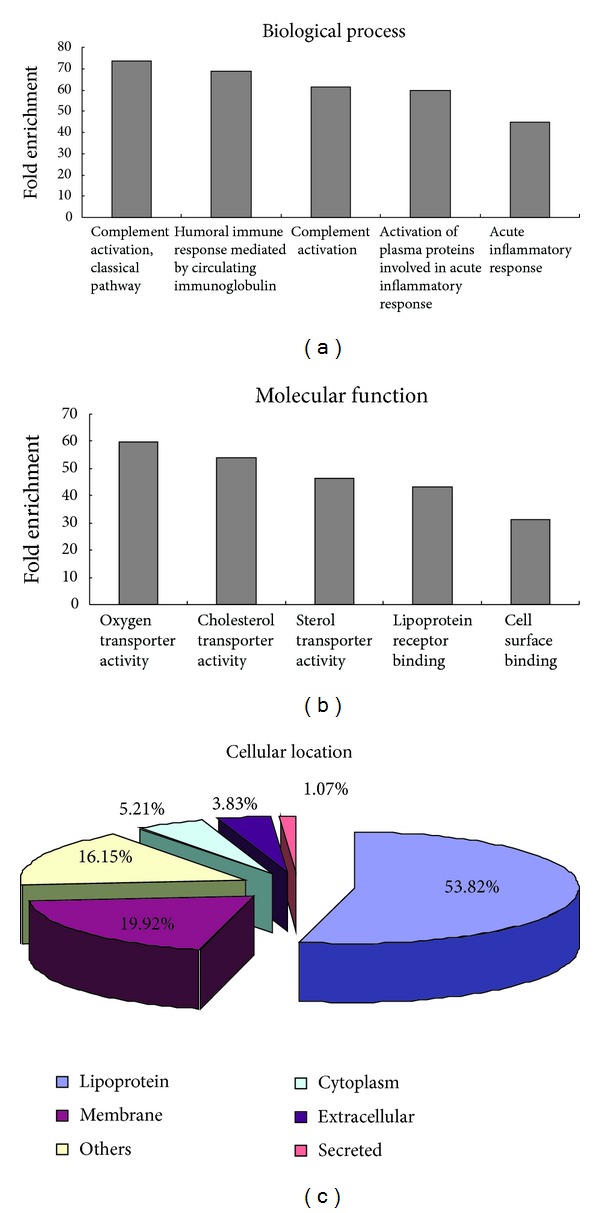
The results of Gene Ontology enrichment analysis. (a) Top 5 canonical biological processes based on the biological process analysis. (b) Top 5 molecular functions based on the molecular function analysis. (c) The cellular component enrichment result.

The top 5 canonical biological processes based on the number of proteins were classical complement activation (*P* = 9.97*E* − 31), humoral immune response mediated by circulating immunoglobulin (*P* = 5.85*E* − 30), complement activation (*P* = 4.36*E* − 35), activation of serum proteins involved in acute inflammatory response (*P* = 8.86*E* − 35), and acute inflammatory response (*P* = 2.87*E* − 54) (Figure 1(a) and Table S1). Molecular function enrichment revealed that the top 5 functions were oxygen transporter activity (*P* = 2.96*E* − 08), cholesterol transporter activity (*P* = 1.54*E* − 06), sterol transporter activity (*P* = 3.08*E* − 06), lipoprotein receptor binding (*P* = 4.18*E* − 06), and cell surface binding (*P* = 7.30*E* − 08) (Figure 1(b) and Table S2). Within the annotated proteins, their location was found to be spread throughout extracellular part and several cellular compartments, with cytoplasmic and membrane proteins being similarly represented. A significant amount of proteins were annotated as lipoproteins (53.82%), whereas 19.92% was classified as membrane and 5.21% as belonging to cytoplasm (Figure 1(c) and Table S3). Cellular signaling pathways enrichment analysis is listed in Tables S4, mapped by proteins with significant abundance changes. Among those 11 pathways generated, the most significant one was about complement and coagulation cascades (*P* = 5.44*E* − 54).

### 3.3. Immunoblot Analysis of Selected Proteins in PF Patient

**Figure 2:**
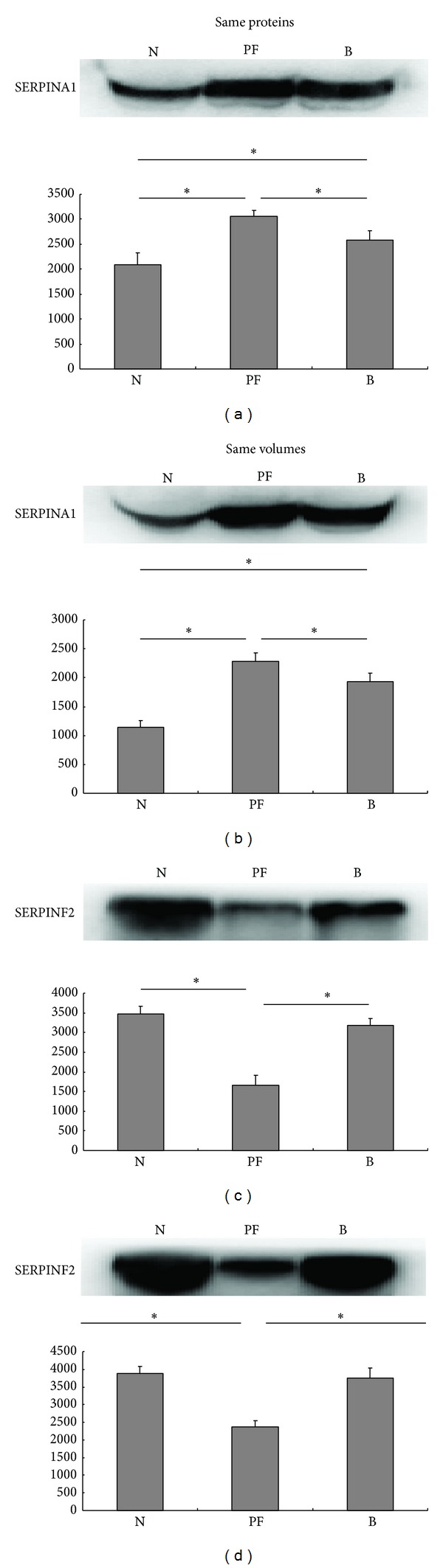
Validation by Western blot of two differentially expressed proteins SERPINA1 and SERPINF2. Serum samples from three healthy individuals (N), one PF patient (PF), and three burn sepsis patients (B) were used to perform WB. (a) and (c) Validation by same proteins WB and histograms visualize normalized mean relative-integrated-density ± standard deviation values. The significance of expression changes was performed by one-way ANOVA test: *P ≤ 0.05. (b) and (d) Validation by same volumes WB and histograms visualize normalized mean relative-integrated-density ± standard deviation values. The significance of expression changes was performed by one-way ANOVA test: *P ≤ 0.05.

To confirm the reliability of the differentially expressed proteins obtained by proteomics, we selected two proteins: SERPINA1 and SERPINF2, which were considered relevant due to their interactions with coagulation cascade, to detect their expression by Western blot. Owing to the lack of proper internal control for serum proteins, we adopted the same loading volumes and same loading proteins ways to compare the changes in serum proteins. We observed an increased SERPINA1 (*P* < 0.05) and decreased SERPINF2 (*P* < 0.05) in the PF patient compared with the healthy individuals and the burn sepsis patients (Figure 2), which was similar to the HPLC-chip/MS results. Moreover, we found an increased SERPINA1 (*P* < 0.05) in the burn sepsis patients compared with the healthy individuals, but there was no significant difference between the two groups about SERPINF2.

## 4. Discussion

PF is a hematological emergency with skin necrosis and DIC. PF patients have increased susceptibility to multiple organ dysfunction syndrome (MODS), and the mortality rate in the acute phase ranges between 18% and 40% [26]. PF can be classified into three forms according to the triggering mechanisms: inherited abnormalities of protein C or other coagulation systems, acute infectious disease, and idiopathic PF [27]. The inherited form occurs mainly in babies and small children, and significant advances have been made in the management of hereditary neonatal PF because of a better understanding of its pathogenesis.

The pathophysiology of infectious PF manifests the full spectrum of the complexity of inflammation and progressive thrombotic disorder. It is deduced that the loss of anticoagulant and anti-inflammatory proteins inhibits fibrinolysis and leads to further activation of inflammatory pathways, which may promote thrombus formation [28, 29]. However, there is no adequate explanation for why the necrotic lesions develop at particular skin sites in conjunction with an acute infectious illness and why not every infectious patient with DIC develops PF. From a therapeutic standpoint, there are no reliable biomarkers available at present for early assessment of PF following infection, so clinicians have been forced to passively accept and treat the disease according to symptoms. And, for many years, fresh frozen plasma (FFP), platelet concentrates, and cryoprecipitate have been remaining central in the treatment of infectious PF [1]. Although additional experimental treatment has been proposed, their efficacy and safety remain experimental, and in many cases controversial [28]. It is conceivable that identification of specific PF biomarkers is of great importance for the PF diagnosis and medical management.

Since the main pathways related to PF were associated with hemostasis, serum would be a proper biological sample to study the disease. Plasma proteomics provides a comprehensive repertoire of experimental techniques for identification as well as quantification of serum proteins. Here, we provided for the first time a relative broad picture of the human serum proteome response to PF. Label-free HPLC-Chip/MS proteomics was applied in this study. Comparing to labeling-based quantification, label-free quantification avoids labeling steps, offers advantages related to the number of samples and the protein detection sensitivity, and has emerged as a high-throughput method in the investigation of scientific questions with clinical relevance over the years [30].

Among the 262 reliably identified proteins, 27 proteins had not been reported with clear implication with burn injury or sepsis [23–25] and might be specifically associated with PF. GO enrichment analysis showed that the observed quantitative changes mainly supported a significant inflammatory activation represented by the complement, humoral immune, and acute inflammation response, such as C1QC, C7, C8G, CFI, and LBP. The complement system has traditionally been considered the first line of innate immune defense against invading pathogens [31]. The increased C1QC reflected the elevated ability of binding antibodies to their target antigens, and the C7 and C8G were closely related to the formation of membrane attack complex. Their decrease may fail to kill the pathogens by complement and lead to inflammatory activation and PF consequently.

In order to validate the results of HPLC-Chip/MS analysis, some identified differentially expressed proteins were further selected and confirmed by Western blot. Considering their critical role in the coagulation cascade, SERPINA1 and SERPINF2 were selected. Western blot proved the increased SERPINA1 and decreased SERPINF2 in the PF patient, which was consistent with the HPLC-Chip/MS results. SERPINA1 is a highly effective inhibitor of neutrophil elastase, plasmin, thrombin, trypsin, chymotrypsin, and plasminogen activator [32–35]. In addition, it is an acute-phase protein and is positively associated with necrosis and inflammation [36]. SERPINF2 is the principal inhibitor of fibrinolysis. Its decrease results not only in uncontrolled fibrinolysis and thrombosis [37] but also in a bleeding disorder through altering the integrity of the extracellular matrix (ECM) [38, 39]. There is increasing evidence that SERPINF2 is a useful alternative target in the development of new therapeutics for thrombotic diseases. Here, the increase of SERPINA1 serum levels reflected the elevated inflammation damage [40] in PF and may serve as a severity index, while the decrease of SERPINF2 was more closely related to DIC during the PF process and may be used as a new therapeutic target for the symptomatic treatment of PF [41].

At last, the main limitation of this present study is that we only had one infectious PF patient, owing to the rarity and randomness of this disease, which may introduce individual variation biases in proteomics analyses. Otherwise, the average age of the healthy control group was 53 ± 6 years old, as well as 55 ± 4 years old of the burn group, while the PF patient was 64 years old. The potential for these alterations to be attributed to age could not be excluded. The outcome of this study needs to be further verified and studied in the future research of these diseases.

## 5. Conclusion

We provided for the first time an overview of serum proteomics changes between healthy individuals and a PF patient. We found 38 differentially expressed proteins in the serum of the PF patient compared with the healthy controls, which mainly participated in acute inflammatory responses, complement activation, immune responses and coagulation cascades. This study provides an initial basis for future studies of PF, and differentially expressed proteins might be new therapeutic targets to these diseases.

## Supplementary Material

S1: The raw result of the Spectrum Mill proteomics analysis.
Supplemental materials: The MS parameters and the database search parameters setting.
Table S1: The raw result of biological processes based on GO analysis by DAVID.
Table S2: The raw result of molecular functions based on GO analysis by DAVID.
Table S3: The raw result of cellular components based on GO analysis by DAVID.
Table S4: The raw result of physiological pathways enrichment analysis by DAVID.

## Figures and Tables

**Table 1:** Patient demographics^a^.

	Control (*n* = 3)	PF (*n* = 1)	Burn sepsis^b^ (*n* = 3)
Age (years)	53 ± 6	64	55 ± 4
Gender (F/M)	3/0	1/0	3/0
Total body surface area burned (%)		2	48 ± 14
Third degree burn (%)		1	13 ± 9
Time since burn injury (day)		18	15 ± 4
Time since sepsis (day)		7	6 ± 3

^a^Data are presented as mean ± standard deviation.

^b^The three burn sepsis serum samples are only applied in Western blot validation.

**Table 2:** List of 27 potentially purpura fulminans-associated proteins (SpC_PF/N_ ≥ 2 or ≤0.5) identified by HPLC-Chip/MS across the two biological groups.

Gene symbol	Protein name	Protein function	% AA coverage	Distinct peptides	SpC_PF/N_	Up-/downregulation
AGT	Angiotensinogen	Maintain blood pressure	18	7	10.32	Up
ALDOB	Fructose-bisphosphate aldolase B	Glycolysis	13	4	38.69	Up
APCS	Serum amyloid P-component	Amyloidosis	16	3	2.06	Up
APOC3	Apolipoprotein C-III	Lipoprotein metabolism	27	2	2.06	Up
APOD	Apolipoprotein D	Lipoprotein metabolism	25	4	2.32	Up
C1QC	Complement C1q subcomponent subunit C	Complement system	17	3	3.1	Up
C7	Complement component 7	Complement system	6	3	0.04	Down
C8G	Complement component 8 gamma	Complement system	23	3	0.04	Down
CFI	Complement factor I	Complement system	6	3	4.64	Up
CPN2	Carboxypeptidase N subunit 2		5	2	6.19	Up
F5	Coagulation factor V	Coagulation cascade	2	4	0.05	Down
FGA	Fibrinogen alpha chain	Coagulation cascade	4	3	67.7	Up
GC	Vitamin D binding protein	Bind vitamin D	25	11	2.58	Up
GSN	Gelsolin	Assembly and disassembly of actin filaments	16	8	0.23	Down
IGFALS	Insulin-like growth factor binding protein complex acid labile chain	Bind insulin-like growth factors	7	3	3.35	Up
LBP	Lipopolysaccharide-binding protein	Immune response	8	3	17.03	Up
LRG1	Leucine-rich alpha-2-glycoprotein 1	Signal transduction	20	6	2.63	Up
LUM	Lumican	Collagen fibril organization	5	2	3.16	Up
ORM2	Alpha-1-acid glycoprotein 2	Acute phase response	21	4	3.41	Up
PGLYRP2	N-acetylmuramoyl-L-alanine amidase	Digest peptidoglycan	4	2	0.02	Down
PKM2	Pyruvate kinase isozymes	Glycolysis	4	2	38.69	Up
SAA	Serum amyloid A protein	Acute phase protein	39	3	58.03	Up
SERPINA1	Alpha-1-antitrypsin	Serine protease inhibitors	20	8	145.08	Up
SERPINA6	Corticosteroid-binding globulin	Transport glucocorticoids	6	2	3.16	Up
SERPINA7	Thyroxine-binding globulin	Transport thyroid hormone	4	2	0.02	Down
SERPINF1	Pigment epithelium derived factor	Inhibit angiogenesis	5	2	29.02	Up
SERPINF2	Alpha-2 antiplasmin	Coagulation cascade	8	3	0.39	Down
