# Supplementary material for: Identification of Differentially Expressed Serum Proteins in Infectious Purpura Fulminans
Source: Dis Markers. 2014 Feb 10;2014:698383. doi: 10.1155/2014/698383 (PMC3934775; doi:10.1155/2014/698383)
Supplement: Supplementary file 1 — S1: The raw result of the Spectrum Mill proteomics analysis. Supplemental materials: The MS parameters and the database search parameters setting. Table S1: The raw result of biological processes based on GO analysis by DAVID. Table S2: The raw result of molecular functions based on GO analysis by DAVID. Table S3: The raw result of cellular components based on GO analysis by DAVID. Table S4: The raw result of physiological pathways enrichment analysis by DAVID. [file 698383.f1.zip › S1.pdf]

Agilent Technologies Agilent Spectrum Mill - Protein/Peptide Summary

Spectrum Mill | Summary Settings | Autovalidation | Easy MS/MS | MS/MS Search | Spectrum Summary | Build TIC | Tool Belt | Help

Display Peptides by Protein SubGroup

Results Shown Filtered by Validation Category: valid

Data Directory: msdataSM\101215TMMU_HJY
hit table read - SpecFeatures read
valid hits read from tagSummary file - **Files:** 2802 **Hits:** 6420
Data Directory: msdataSM\101220TMMU_HJY
hit table read - SpecFeatures read
valid hits read from tagSummary file - **Files:** 1911 **Hits:** 4152
beginning to assemble proteins .... proteins assembled 1.671875 sec
proteins filtered by unique peptides 0.496167 sec
proteins filtered by score
calculated protein coverage maps 2.159719 sec
beginning to roll up proteins into groups ... . proteins rolled up into groups 1.656199 sec
protein groups ready for display
proteinGroupingMethod: oneSharedPeptide
Mean intensity: sum of intensity for all spectra of peptides belonging to protein / # spectra

101215TMMU_HJY # spectra mean intensity	101220TMMU_HJY # spectra mean intensity	Protein MW (Da)	Protein pI	Species	Database Accession #	%AA Coverage	Distinct Peptides (#)	Distinct Summed MS/MS Search Score	Group #	Protein Name
311<br>4.71e+008	198<br>1.53e+008	187149.1	6.02	HUMAN	IPI00783987	47	68	1057.54	☐<br>1.1	C3 Complement C3 precursor (Fragment)
283<br>5.11e+008	154<br>1.71e+008	144810.1	7.28	HUMAN	IPI00887739	48	54	853.24	☐	LOC100133511 hypothetical protein, partial
20<br>6.46e+007	28<br>8.08e+007	44929.9	4.94	HUMAN	IPI00739237	37	12	167.77	☐	LOC653879 similar to complement component 3
449<br>5.86e+008	282<br>1.02e+009	163278.8	6.00	HUMAN	IPI00478003	47	51	820.26	☐<br>2.1	A2M Alpha-2-macroglobulin precursor
105<br>6.57e+008	41<br>4.24e+008	163832.8	5.97	HUMAN	IPI00025426	6	8	129.13	☐	PZP Isoform 1 of Pregnancy zone protein precursor
105<br>6.57e+008	41<br>4.24e+008	140368.5	5.90	HUMAN	IPI00748437	7	8	129.13	☐	PZP Uncharacterized protein PZP
105<br>6.57e+008	41<br>4.24e+008	140364.5	5.90	HUMAN	IPI00884981	7	8	129.13	☐	PZP Isoform 2 of Pregnancy zone protein precursor
27<br>4.02e+008	36<br>5.60e+008	18762.7	8.77	HUMAN	IPI00789547	45	6	83.02	☐	A2M 19 kDa protein
4<br>4.11e+008	1<br>4.46e+007	18723.9	6.08	HUMAN	IPI00878729	29	3	43.60	☐	A2M 19 kDa protein
17<br>1.11e+008	7<br>1.09e+008	12992.8	6.08	HUMAN	IPI00796830	42	3	41.27	☐	A2M 13 kDa protein
160<br>5.44e+008	101<br>1.41e+008	515565.2	6.61	HUMAN	IPI00022229	13	47	719.71	☐<br>3.1	APOB Apolipoprotein B-100 precursor
98<br>2.45e+008	83<br>7.72e+007	192752.5	6.89	HUMAN	IPI00418163	26	36	551.13	☐<br>4.1	C4B complement component 4B preproprotein
98<br>2.45e+008	83<br>7.72e+007	192749.5	6.89	HUMAN	IPI00887154	26	36	551.13	☐	DADB-112B14.11 Complement component 4B
103<br>2.39e+008	92<br>7.81e+007	192743.5	6.66	HUMAN	IPI00643525	24	35	537.05	☐	C4A Complement component 4A
98<br>2.54e+008	90<br>7.75e+007	192794.5	6.74	HUMAN	IPI00654875	24	34	520.20	☐	C4B Complement C4-B precursor
101<br>2.31e+008	91<br>7.88e+007	192786.5	6.66	HUMAN	IPI00889723	24	34	519.73	☐	C4A complement component 4A preproprotein
95<br>2.43e+008	90<br>7.75e+007	192772.5	6.66	HUMAN	IPI00032258	22	33	504.01	☐	C4A Complement C4-A precursor
7<br>1.94e+008	4<br>1.70e+008	15242.5	8.29	HUMAN	IPI00843913	33	4	57.42	☐	C4A ZA protein

345<br>4.27e+009	210<br>3.97e+009	30778.0	5.56	HUMAN	IPI00021841	59	22	372.47	☐<br>5.1	APOA1 Apolipoprotein A-I precursor
298<br>4.87e+009	181<br>4.60e+009	27908.7	5.80	HUMAN	IPI00853525	50	17	280.64	☐	APOA1 Apolipoprotein A1
70<br>3.39e+008	30<br>2.12e+008	139097.3	6.21	HUMAN	IPI00029739	27	26	366.70	☐<br>6.1	CFH Isoform 1 of Complement factor H precursor
24<br>1.76e+008	11<br>5.82e+007	51034.2	6.77	HUMAN	IPI00218999	26	9	118.79	☐	CFH Isoform 2 of Complement factor H precursor
19<br>1.66e+008	9<br>4.81e+007	43846.0	7.67	HUMAN	IPI00515041	25	8	104.61	☐	CFH Uncharacterized protein CFH
1<br>1.48e+006	0<br>0.00e+000	37323.5	7.72	HUMAN	IPI00027507	3	1	11.56	☐	CFHR3 Complement factor H-related protein 3 precursor
1<br>1.48e+006	0<br>0.00e+000	26120.0	8.37	HUMAN	IPI00844262	4	1	11.56	☐	CFHR3 CFHR3 protein
1<br>1.48e+006	0<br>0.00e+000	22240.5	7.92	HUMAN	IPI00654723	5	1	11.56	☐	CFHR3 CFHR3 protein
139<br>1.02e+009	99<br>6.17e+008	51676.7	6.55	HUMAN	IPI00022488	51	21	318.14	☐<br>7.1	HPX Hemopexin precursor
57<br>2.38e+008	10<br>4.43e+008	262607.9	5.45	HUMAN	IPI00022418	12	19	267.96	☐<br>8.1	FN1 Isoform 1 of Fibronectin precursor
57<br>2.38e+008	10<br>4.43e+008	259199.2	5.47	HUMAN	IPI00339223	12	19	267.96	☐	FN1 Isoform 3 of Fibronectin precursor
57<br>2.38e+008	10<br>4.43e+008	256513.2	5.53	HUMAN	IPI00414283	12	19	267.96	☐	FN1 fibronectin 1 isoform 4 preproprotein
57<br>2.38e+008	10<br>4.43e+008	252794.2	5.61	HUMAN	IPI00339228	12	19	267.96	☐	FN1 Isoform 8 of Fibronectin precursor
57<br>2.38e+008	10<br>4.43e+008	249385.5	5.64	HUMAN	IPI00855777	12	19	267.96	☐	FN1 Isoform 14 of Fibronectin precursor
57<br>2.38e+008	10<br>4.43e+008	243317.5	5.48	HUMAN	IPI00339225	13	19	267.96	☐	FN1 Isoform 5 of Fibronectin precursor
57<br>2.38e+008	10<br>4.43e+008	239609.4	5.58	HUMAN	IPI00479723	13	19	267.96	☐	FN1 Isoform 10 of Fibronectin precursor
56<br>2.27e+008	10<br>4.43e+008	272303.7	5.30	HUMAN	IPI00855785	10	18	256.86	☐	FN1 Isoform 15 of Fibronectin precursor
56<br>2.27e+008	10<br>4.43e+008	268895.0	5.32	HUMAN	IPI00339227	10	18	256.86	☐	FN1 Isoform 7 of Fibronectin precursor
56<br>2.27e+008	10<br>4.43e+008	266209.0	5.36	HUMAN	IPI00845263	10	18	256.86	☐	FN1 fibronectin 1 isoform 2 preproprotein
56<br>2.27e+008	10<br>4.43e+008	262389.7	5.37	HUMAN	IPI00339319	11	18	256.86	☐	FN1 Isoform 11 of Fibronectin precursor
56<br>2.27e+008	10<br>4.43e+008	249305.3	5.39	HUMAN	IPI00867588	11	18	256.86	☐	FN1 Isoform 13 of Fibronectin precursor
54<br>2.32e+008	10<br>4.43e+008	221274.8	5.78	HUMAN	IPI00556632	12	17	244.13	☐	FN1 Isoform 12 of Fibronectin precursor
49<br>2.44e+008	10<br>4.43e+008	262554.9	5.38	HUMAN	IPI00873210	10	17	237.72	☐	FN1 263 kDa protein
47<br>2.71e+008	9<br>4.86e+008	240478.2	5.44	HUMAN	IPI00339226	11	17	236.29	☐	FN1 Isoform 6 of Fibronectin precursor
44<br>2.84e+008	9<br>4.86e+008	222944.9	5.43	HUMAN	IPI00339224	12	16	220.27	☐	FN1 Isoform 4 of Fibronectin precursor
11<br>2.10e+008	1<br>5.54e+007	37341.2	6.66	HUMAN	IPI00856050	9	2	28.57	☐	- Fibronectin splice variant F (Fragment)
4<br>2.23e+008	0<br>0.00e+000	71943.6	6.57	HUMAN	IPI00411462	4	2	25.83	☐	FN1 Isoform 2 of Fibronectin precursor
101<br>4.97e+008	71<br>4.57e+008	122205.8	5.44	HUMAN	IPI00017601	19	15	233.04	☐<br>9.1	CP Ceruloplasmin precursor

62<br>3.32e+008	48<br>1.08e+008	97069.2	5.31	HUMAN	IPI00794184	15	9	141.10	☐	CP 97 kDa protein
41<br>4.52e+008	23<br>1.14e+008	20193.9	5.27	HUMAN	IPI00793108	28	3	54.89	☐	CP 20 kDa protein
7<br>7.03e+007	0<br>0.00e+000	20030.9	7.10	HUMAN	IPI00879084	14	2	24.64	☐	CP 20 kDa protein
87<br>8.21e+008	123<br>2.09e+008	50598.5	5.42	HUMAN	IPI00550991	30	14	210.45	☐<br>10.1	SERPINA3 Alpha-1-antichymotrypsin precursor
87<br>8.21e+008	123<br>2.09e+008	47651.1	5.33	HUMAN	IPI00847635	31	14	210.45	☐	SERPINA3 Isoform 1 of Alpha-1-antichymotrypsin precursor
28<br>4.58e+008	21<br>1.44e+008	45399.3	5.28	HUMAN	IPI00304273	37	14	203.47	☐<br>11.1	APOA4 Apolipoprotein A-IV precursor
28<br>4.58e+008	21<br>1.44e+008	45372.2	5.28	HUMAN	IPI00847179	37	14	203.47	☐	APOA4 apolipoprotein A-IV precursor
41<br>4.52e+008	18<br>6.89e+007	47901.5	6.29	HUMAN	IPI00797833	37	14	193.16	☐<br>12.1	KNG1 Kininogen 1
40<br>4.63e+008	18<br>6.89e+007	71957.8	6.34	HUMAN	IPI00032328	22	13	184.44	☐	KNG1 Isoform HMW of Kininogen-1 precursor
40<br>4.63e+008	18<br>6.89e+007	47883.5	6.29	HUMAN	IPI00215894	34	13	184.44	☐	KNG1 Isoform LMW of Kininogen-1 precursor
35<br>5.23e+008	14<br>5.22e+007	33085.6	6.27	HUMAN	IPI00789376	36	10	153.01	☐	KNG1 KNG1 protein
5<br>4.18e+007	4<br>1.27e+008	17352.6	4.83	HUMAN	IPI00797097	25	3	31.43	☐	KNG1 17 kDa protein
28<br>4.85e+008	11<br>3.76e+007	188306.3	6.11	HUMAN	IPI00032291	10	13	176.86	☐<br>13.1	C5 Complement C5 precursor
6<br>9.07e+006	5<br>2.24e+007	123352.0	8.43	HUMAN	IPI00816741	8	7	84.75	☐	C5 Complement component 5 variant (Fragment)
18<br>7.66e+007	30<br>7.74e+007	52917.9	5.32	HUMAN	IPI00742696	25	11	161.86	☐<br>14.1	GC vitamin D-binding protein precursor
17<br>7.01e+007	27<br>7.00e+007	52964.0	5.40	HUMAN	IPI00555812	22	9	134.01	☐	GC Vitamin D-binding protein precursor
81<br>7.39e+008	22<br>4.07e+008	52691.8	6.12	HUMAN	IPI00032179	27	10	158.91	☐<br>15.1	SERPINC1 Antithrombin III variant
44<br>6.12e+008	6<br>5.86e+008	29092.6	9.03	HUMAN	IPI00844156	20	4	65.89	☐	SERPINC1 SERPINC1 protein
38<br>2.23e+008	12<br>1.37e+008	90569.6	7.04	HUMAN	IPI00019580	19	11	153.65	☐<br>16.1	PLG Plasminogen precursor
74<br>4.55e+008	20<br>1.03e+008	59578.6	7.09	HUMAN	IPI00022371	27	10	152.35	☐<br>17.1	HRG Histidine-rich glycoprotein precursor
34<br>3.15e+008	6<br>1.29e+008	70037.3	5.64	HUMAN	IPI00019568	22	10	148.29	☐<br>18.1	F2 Prothrombin precursor (Fragment)
16<br>2.06e+008	2<br>4.92e+007	35931.8	4.74	HUMAN	IPI00877967	15	3	51.54	☐	F2 36 kDa protein
27<br>1.80e+008	17<br>2.13e+008	55154.5	6.09	HUMAN	IPI00291866	20	9	147.70	☐<br>19.1	SERPING1 Plasma protease C1 inhibitor precursor
23<br>1.88e+008	16<br>2.24e+008	37288.2	7.91	HUMAN	IPI00556459	27	8	133.67	☐	SERPING1 Serine/cysteine proteinase inhibitor clade G member 1 splice variant 2 (Fragment)
25<br>1.89e+008	16<br>2.23e+008	59492.4	6.28	HUMAN	IPI00879931	16	8	127.12	☐	SERPING1 cDNA FLJ78023, highly similar to Homo sapiens serine (or cysteine) proteinase inhibitor, clade G (C1inhibitor), member 1, (angioedema, hereditary) (SERPING1), mRNA
4<br>1.33e+008	1<br>4.69e+007	18153.4	4.48	HUMAN	IPI00877698	5	1	14.03	☐	SERPING1 18 kDa protein
4<br>1.33e+008	1<br>4.69e+007	11785.3	4.94	HUMAN	IPI00385045	8	1	14.03	☐	SERPING1 C1 inhibitor mutant (Fragment)

28<br>2.40e+008	10<br>3.87e+008	103325.9	6.51	HUMAN	IPI00294193	11	10	140.65	☐<br>20.1	ITIH4 Isoform 1 of Inter-alpha-trypsin inhibitor heavy chain H4 precursor
27<br>2.49e+008	10<br>3.87e+008	101209.5	6.21	HUMAN	IPI00218192	10	9	132.43	☐	ITIH4 Isoform 2 of Inter-alpha-trypsin inhibitor heavy chain H4 precursor
27<br>2.49e+008	10<br>3.87e+008	84248.7	5.31	HUMAN	IPI00878609	12	9	132.43	☐	ITIH4 Protein
14<br>2.08e+008	5<br>3.68e+008	70953.5	5.43	HUMAN	IPI00878152	10	6	88.78	☐	ITIH4 71 kDa protein
11<br>1.92e+008	5<br>4.06e+008	15749.3	9.49	HUMAN	IPI00556036	7	2	34.93	☐	ITIH4 Inter-alpha (Globulin) inhibitor H4 (Plasma Kallikrein-sensitive glycoprotein) variant
20<br>1.93e+009	22<br>3.12e+008	85533.4	6.67	HUMAN	IPI00019591	17	9	140.04	☐<br>21.1	CFB Isoform 1 of Complement factor B precursor (Fragment)
12<br>1.98e+008	15<br>1.31e+008	85534.3	6.44	HUMAN	IPI00639937	15	8	125.77	☐	CFB Complement factor B
5<br>3.27e+008	11<br>1.47e+008	68872.9	6.11	HUMAN	IPI00218508	7	3	54.90	☐	CFB Isoform 2 of Complement factor B precursor (Fragment)
41<br>2.63e+008	6<br>1.12e+008	85697.9	5.90	HUMAN	IPI00026314	16	8	128.28	☐<br>22.1	GSN Isoform 1 of Gelsolin precursor
41<br>2.63e+008	6<br>1.12e+008	80641.0	5.58	HUMAN	IPI00646773	17	8	128.28	☐	GSN Isoform 2 of Gelsolin precursor
30<br>2.09e+008	4<br>1.27e+008	52373.1	5.21	HUMAN	IPI00513782	21	6	90.03	☐	GSN Gelsolin
17<br>1.68e+008	3<br>1.31e+008	22948.9	4.60	HUMAN	IPI00796316	25	3	54.22	☐	GSN Gelsolin
17<br>1.68e+008	3<br>1.31e+008	20783.6	4.69	HUMAN	IPI00377087	28	3	54.22	☐	GSN Gelsolin
11<br>4.11e+008	2<br>8.21e+007	28953.8	7.71	HUMAN	IPI00641047	10	2	38.25	☐	GSN Gelsolin
11<br>4.11e+008	2<br>8.21e+007	25857.3	6.61	HUMAN	IPI00647556	11	2	38.25	☐	GSN Gelsolin
41<br>3.74e+008	15<br>2.82e+008	54272.8	5.58	HUMAN	IPI00022895	21	9	125.60	☐<br>23.1	A1BG Alpha-1B-glycoprotein precursor
37<br>3.90e+008	14<br>2.67e+008	54253.8	5.56	HUMAN	IPI00745089	19	8	111.16	☐	A1BG alpha 1B-glycoprotein precursor
29<br>4.79e+008	11<br>2.80e+008	40718.1	5.50	HUMAN	IPI00644018	17	5	75.99	☐	A1BG 41 kDa protein
28<br>4.89e+008	11<br>2.80e+008	35271.0	5.96	HUMAN	IPI00646799	15	4	67.58	☐	A1BG Putative uncharacterized protein DKFZp686F0970
32<br>1.62e+008	5<br>3.15e+008	106437.0	6.40	HUMAN	IPI00305461	12	9	124.57	☐<br>24.1	ITIH2 Inter-alpha-trypsin inhibitor heavy chain H2 precursor
32<br>1.62e+008	5<br>3.15e+008	105216.6	6.56	HUMAN	IPI00645038	12	9	124.57	☐	ITIH2 Inter-alpha (Globulin) inhibitor H2
14<br>5.53e+007	3<br>5.16e+008	26821.0	9.08	HUMAN	IPI00514159	23	4	60.09	☐	ITIH2 Inter-alpha (Globulin) inhibitor H2
6<br>1.69e+008	40<br>5.79e+007	53154.5	5.87	HUMAN	IPI00032220	18	7	123.11	☐<br>25.1	AGT Angiotensinogen precursor
31<br>1.64e+008	23<br>5.05e+007	38999.7	5.95	HUMAN	IPI00022426	30	8	118.30	☐<br>26.1	AMBP AMBP protein precursor
15<br>2.08e+008	13<br>1.25e+008	36154.3	5.65	HUMAN	IPI00021842	31	7	112.54	☐<br>27.1	APOE Apolipoprotein E precursor
4<br>1.09e+008	4<br>1.52e+008	32558.2	6.99	HUMAN	IPI00878953	21	4	64.76	☐	APOE MRNA for apolipoprotein E
2<br>1.78e+008	1<br>8.94e+007	24903.6	5.75	HUMAN	IPI00879456	21	3	47.73	☐	APOE 25 kDa protein
2<br>1.78e+008	1<br>8.94e+007	24647.3	5.75	HUMAN	IPI00879368	21	3	47.73	☐	APOE Apolipoprotein E

23<br>6.30e+008	10<br>1.89e+008	101389.7	6.31	HUMAN	IPI00292530	10	6	110.31	☐<br>28.1	ITIH1 Inter-alpha-trypsin inhibitor heavy chain H1 precursor
1<br>1.72e+009	0<br>0.00e+000	55367.7	5.91	HUMAN	IPI00383338	5	1	17.05	☐	ITIH1 PRO2769
0<br>0.00e+000	15<br>8.01e+007	46736.8	5.37	HUMAN	IPI00553177	20	8	108.17	☐<br>29.1	SERPINA1 Isoform 1 of Alpha-1-antitrypsin precursor
0<br>0.00e+000	11<br>6.68e+007	40263.0	5.26	HUMAN	IPI00790784	19	6	81.74	☐	SERPINA1 Isoform 2 of Alpha-1-antitrypsin precursor
0<br>0.00e+000	9<br>5.73e+007	13097.5	8.93	HUMAN	IPI00305457	35	4	57.00	☐	SERPINA1 PRO2275
0<br>0.00e+000	6<br>1.14e+008	34755.6	5.04	HUMAN	IPI00869004	13	4	51.17	☐	SERPINA1 Isoform 3 of Alpha-1-antitrypsin precursor
48<br>1.86e+009	42<br>2.33e+008	23539.7	5.02	HUMAN	IPI00884926	33	6	104.40	☐<br>30.1	ORM1 orosomucoid 1 precursor
48<br>1.86e+009	42<br>2.33e+008	23511.7	4.93	HUMAN	IPI00022429	33	6	104.40	☐	ORM1 Alpha-1-acid glycoprotein 1 precursor
10<br>1.44e+009	22<br>5.87e+008	23602.8	5.03	HUMAN	IPI00020091	21	4	61.22	☐	ORM2 Alpha-1-acid glycoprotein 2 precursor
34<br>6.22e+008	19<br>8.46e+008	54305.9	5.55	HUMAN	IPI00298971	16	7	101.77	☐<br>31.1	VTN Vitronectin precursor
0<br>0.00e+000	13<br>2.64e+008	15998.5	6.74	HUMAN	IPI00654755	59	7	98.54	☐<br>32.1	HBB Hemoglobin subunit beta
0<br>0.00e+000	10<br>3.12e+008	18930.9	6.28	HUMAN	IPI00884107	36	5	66.75	☐	HBB Beta-globin gene from a thalassemia patient
0<br>0.00e+000	9<br>3.46e+008	11504.2	5.90	HUMAN	IPI00796636	45	4	56.27	☐	HBB Hemoglobin (Fragment)
0<br>0.00e+000	9<br>3.76e+008	16055.6	7.84	HUMAN	IPI00473011	25	3	41.46	☐	HBD;HBB Hemoglobin subunit delta
0<br>0.00e+000	8<br>3.87e+008	11459.1	6.17	HUMAN	IPI00829896	37	3	37.50	☐	HBD Hemoglobin Lepore-Baltimore (Fragment)
0<br>0.00e+000	7<br>4.40e+008	19009.0	7.70	HUMAN	IPI00830113	15	2	26.78	☐	HBB 19 kDa protein
0<br>0.00e+000	7<br>4.40e+008	15419.8	7.74	HUMAN	IPI00657660	18	2	26.78	☐	HBB Hemoglobin delta-beta fusion protein
0<br>0.00e+000	7<br>4.40e+008	11346.0	5.89	HUMAN	IPI00791558	25	2	26.78	☐	HBB Delta-hemoglobin
0<br>0.00e+000	7<br>4.40e+008	9117.5	6.90	HUMAN	IPI00884436	29	2	26.78	☐	- Similar to Beta-hemoglobin
0<br>0.00e+000	6<br>2.19e+008	17429.1	6.04	HUMAN	IPI00749035	6	1	12.53	☐	HBG2 Gamma-G globin
0<br>0.00e+000	6<br>2.19e+008	17316.0	6.04	HUMAN	IPI00744503	6	1	12.53	☐	HBG1 17 kDa protein
0<br>0.00e+000	6<br>2.19e+008	16594.0	6.41	HUMAN	IPI00816618	6	1	12.53	☐	HBG2 Hemoglobin gamma-G (Fragment)
0<br>0.00e+000	6<br>2.19e+008	16202.9	8.67	HUMAN	IPI00217471	6	1	12.53	☐	HBE1 Hemoglobin subunit epsilon
0<br>0.00e+000	6<br>2.19e+008	16140.5	6.65	HUMAN	IPI00220706	6	1	12.53	☐	HBG1 Hemoglobin subunit gamma-1
0<br>0.00e+000	6<br>2.19e+008	16126.5	6.65	HUMAN	IPI00554676	6	1	12.53	☐	HBG2;HBG1 Hemoglobin subunit gamma-2
0<br>0.00e+000	6<br>2.19e+008	15319.1	9.48	HUMAN	IPI00657911	7	1	12.53	☐	HBG2 Gamma-globin
0<br>0.00e+000	6<br>2.19e+008	9470.0	9.10	HUMAN	IPI00853641	11	1	12.53	☐	HBE1 Uncharacterized protein HBE1
0<br>0.00e+000	6<br>2.19e+008	4508.3	9.52	HUMAN	IPI00815947	22	1	12.53	☐	HBB Truncated beta-globin (Fragment)

0<br>0.00e+000	1<br>8.40e+006	3500.1	6.90	HUMAN	IPI00816644	45	1	10.48	☐	HBB Hemoglobin beta chain (Fragment)
10<br>2.47e+008	17<br>1.36e+008	38178.1	6.45	HUMAN	IPI00022417	20	6	93.89	☐<br>33.1	LRG1 Leucine-rich alpha-2-glycoprotein precursor
21<br>3.36e+008	22<br>2.25e+008	69069.6	5.64	HUMAN	IPI00019943	15	7	88.54	☐<br>34.1	AFM Afamin precursor
8<br>4.69e+008	6<br>6.33e+007	63173.8	5.43	HUMAN	IPI00022395	9	5	88.06	☐<br>35.1	C9 Complement component C9 precursor
9<br>7.68e+007	8<br>9.31e+007	80174.1	5.89	HUMAN	IPI00296165	12	6	87.05	☐<br>36.1	C17orf13;ACYP1;C1R Complement C1r subcomponent precursor
2<br>2.14e+007	1<br>2.74e+007	26299.8	5.13	HUMAN	IPI00791901	11	2	23.40	☐	C1R 26 kDa protein
15<br>1.23e+008	12<br>7.40e+007	60178.6	6.72	HUMAN	IPI00292950	14	7	84.55	☐<br>37.1	SERPIND1 Serpin peptidase inhibitor, clade D (Heparin cofactor), member 1
12<br>1.42e+008	12<br>7.40e+007	57070.9	6.41	HUMAN	IPI00879573	11	6	75.07	☐	SERPIND1 Heparin cofactor 2 precursor
15<br>2.65e+008	20<br>2.32e+008	38298.4	8.34	HUMAN	IPI00298828	26	6	82.74	☐<br>38.1	APOH Beta-2-glycoprotein 1 precursor
6<br>1.33e+009	14<br>2.54e+008	20198.9	5.16	HUMAN	IPI00855916	32	5	76.54	☐<br>39.1	- Transthyretin
6<br>1.33e+009	14<br>2.54e+008	15887.1	5.52	HUMAN	IPI00022432	40	5	76.54	☐	TTR Transthyretin precursor
6<br>1.33e+009	8<br>3.25e+008	13154.9	5.34	HUMAN	IPI00646384	30	3	42.60	☐	TTR 13 kDa protein
18<br>7.00e+008	9<br>2.62e+008	57832.9	6.25	HUMAN	IPI00400826	13	4	75.43	☐<br>40.1	CLU clusterin isoform 1
18<br>7.00e+008	9<br>2.62e+008	52494.9	5.89	HUMAN	IPI00291262	15	4	75.43	☐	CLU Clusterin precursor
18<br>7.00e+008	9<br>2.62e+008	52365.8	5.98	HUMAN	IPI00795633	15	4	75.43	☐	CLU CLU
6<br>9.96e+007	3<br>4.81e+007	53510.1	6.52	HUMAN	IPI00793848	6	2	35.72	☐	CLU 54 kDa protein
14<br>1.35e+008	9<br>3.75e+008	39324.9	5.43	HUMAN	IPI00022431	16	4	64.89	☐<br>41.1	AHSG Alpha-2-HS-glycoprotein precursor
1<br>6.57e+008	5<br>6.36e+008	28534.6	4.77	HUMAN	IPI00795830	15	3	44.26	☐	AHSG 29 kDa protein
3<br>1.27e+008	2<br>6.05e+007	71370.2	8.60	HUMAN	IPI00654888	8	4	61.20	☐<br>42.1	KLKB1 Plasma kallikrein precursor
1<br>2.71e+007	0<br>0.00e+000	16620.4	9.04	HUMAN	IPI00879718	12	1	11.48	☐	KLKB1 17 kDa protein
2<br>8.95e+006	3<br>2.13e+007	21275.7	5.06	HUMAN	IPI00006662	25	4	59.69	☐<br>43.1	APOD Apolipoprotein D precursor
0<br>0.00e+000	6<br>3.33e+007	13532.1	6.28	HUMAN	IPI00552578	39	3	56.51	☐<br>44.1	SAA2;SAA1 Serum amyloid A protein precursor
0<br>0.00e+000	5<br>3.16e+007	13527.2	9.20	HUMAN	IPI00006146	22	2	34.69	☐	SAA2;SAA1 serum amyloid A2
1<br>2.97e+007	11<br>4.71e+007	53383.9	6.23	HUMAN	IPI00032311	8	3	55.15	☐<br>45.1	LBP Lipopolysaccharide-binding protein precursor
0<br>0.00e+000	5<br>7.92e+007	67033.6	7.15	HUMAN	IPI00021727	6	3	52.51	☐<br>46.1	C4BPA C4b-binding protein alpha chain precursor
0<br>0.00e+000	3<br>1.12e+008	45907.3	6.10	HUMAN	IPI00872510	6	2	37.40	☐	C4BPA Uncharacterized protein C4BPA
15<br>4.01e+008	5<br>2.65e+008	34258.9	5.71	HUMAN	IPI00166729	14	4	50.52	☐<br>47.1	AZGP1 alpha-2-glycoprotein 1, zinc

5<br>1.46e+008	0<br>0.00e+000	23902.9	5.78	HUMAN	IPI00871596	12	3	33.94	☐	AZGP1 Uncharacterized protein AZGP1 (Fragment)
3<br>1.14e+007	0<br>0.00e+000	18707.4	8.99	HUMAN	IPI00816309	12	2	22.39	☐	AZGP1 AZGP1 protein (Fragment)
7<br>1.49e+008	0<br>0.00e+000	22277.6	8.49	HUMAN	IPI00011261	23	3	48.92	☐<br>48.1	C8G Complement component C8 gamma chain precursor
6<br>1.68e+007	0<br>0.00e+000	17557.3	9.82	HUMAN	IPI00513935	20	2	28.76	☐	C8G Complement component 8, gamma polypeptide
0<br>0.00e+000	4<br>9.62e+007	39473.2	8.00	HUMAN	IPI00218407	13	4	48.66	☐<br>49.1	ALDOB Fructose-bisphosphate aldolase B
0<br>0.00e+000	2<br>1.47e+008	24128.8	6.65	HUMAN	IPI00513830	11	2	27.40	☐	ALDOB Fructose-bisphosphate aldolase
3<br>1.41e+008	6<br>2.13e+007	25773.8	8.61	HUMAN	IPI00022394	17	3	48.37	☐<br>50.1	C1QC Complement C1q subcomponent subunit C precursor
0<br>0.00e+000	9<br>1.80e+008	15280.7	8.72	HUMAN	IPI00853068	25	3	48.23	☐<br>51.1	HBA1;HBA2 Alpha 2 globin variant (Fragment)
0<br>0.00e+000	9<br>1.80e+008	15257.6	8.72	HUMAN	IPI00410714	25	3	48.23	☐	HBA1;HBA2 Hemoglobin subunit alpha
6<br>2.08e+008	13<br>5.92e+007	66035.3	6.33	HUMAN	IPI00020996	7	3	47.84	☐<br>52.1	IGFALS Insulin-like growth factor-binding protein complex acid labile chain precursor
3<br>6.79e+008	1<br>3.19e+006	22567.0	5.52	HUMAN	IPI00009028	20	3	45.84	☐<br>53.1	CLEC3B Tetranectin precursor
2<br>7.46e+008	1<br>3.19e+006	17794.2	4.96	HUMAN	IPI00792115	13	2	29.30	☐	CLEC3B Putative uncharacterized protein DKFZp686H17246
1<br>1.07e+007	1<br>3.19e+006	10656.4	8.38	HUMAN	IPI00791350	9	1	12.12	☐	CLEC3B 11 kDa protein
6<br>9.85e+007	0<br>0.00e+000	93518.9	6.09	HUMAN	IPI00296608	6	3	45.14	☐<br>54.1	C7 Complement component C7 precursor
1<br>9.21e+008	3<br>2.39e+006	65721.7	7.32	HUMAN	IPI00872555	6	3	44.37	☐<br>55.1	CFI cDNA FLJ76262, highly similar to Homo sapiens I factor (complement) (IF), mRNA
1<br>9.21e+008	3<br>2.39e+006	65720.7	7.72	HUMAN	IPI00291867	6	3	44.37	☐	CFI Complement factor I precursor
1<br>9.21e+008	2<br>1.53e+006	42576.8	8.41	HUMAN	IPI00795153	6	2	30.47	☐	CFI 43 kDa protein
1<br>9.21e+008	2<br>1.53e+006	42462.6	8.49	HUMAN	IPI00794070	6	2	30.47	☐	CFI CFI protein
0<br>0.00e+000	1<br>4.10e+006	42663.8	5.73	HUMAN	IPI00796990	3	1	13.90	☐	CFI Light chain of factor I
0<br>0.00e+000	4<br>5.54e+007	57156.4	7.95	HUMAN	IPI00472345	8	3	43.17	☐<br>56.1	IGHG3 IGHG3 protein
0<br>0.00e+000	4<br>5.54e+007	57019.9	8.39	HUMAN	IPI00418153	8	3	43.17	☐	IGHM Putative uncharacterized protein DKFZp686I15212
0<br>0.00e+000	4<br>5.54e+007	56813.6	6.43	HUMAN	IPI00784894	8	3	43.17	☐	- Putative uncharacterized protein
0<br>0.00e+000	4<br>5.54e+007	56111.3	7.80	HUMAN	IPI00168728	8	3	43.17	☐	IGHM FLJ00385 protein (Fragment)
0<br>0.00e+000	4<br>5.54e+007	49135.3	6.50	HUMAN	IPI00829850	9	3	43.17	☐	IGHG3 Protein
0<br>0.00e+000	4<br>5.54e+007	41329.3	8.23	HUMAN	IPI00827754	11	3	43.17	☐	IGHG3 C gamma 3
0<br>0.00e+000	4<br>5.54e+007	39720.4	8.30	HUMAN	IPI00829716	12	3	43.17	☐	IGHG3 Uncharacterized protein IGHG3 (Fragment)
0<br>0.00e+000	4<br>5.54e+007	38111.5	8.38	HUMAN	IPI00829940	12	3	43.17	☐	IGHG3 Uncharacterized protein IGHG3 (Fragment)
0<br>0.00e+000	3<br>3.62e+007	39125.8	7.87	HUMAN	IPI00830033	8	2	31.49	☐	IGHM Full-length cDNA clone CS0DI019YF20 of Placenta of Homo sapiens (Fragment)

0<br>0.00e+000	3<br>7.33e+007	60102.5	7.48	HUMAN	IPI00448925	4	2	26.84	☐	IGHG1 IGHG1 protein
0<br>0.00e+000	3<br>7.33e+007	52852.3	8.74	HUMAN	IPI00384938	4	2	26.84	☐	IGHG1 Putative uncharacterized protein DKFZp686N02209
0<br>0.00e+000	3<br>7.33e+007	52759.2	8.75	HUMAN	IPI00423466	4	2	26.84	☐	IGHG1 Putative uncharacterized protein DKFZp686H20196
0<br>0.00e+000	3<br>7.33e+007	52666.9	7.50	HUMAN	IPI00472610	5	2	26.84	☐	IGHM IGHM protein
0<br>0.00e+000	3<br>7.33e+007	52612.9	8.46	HUMAN	IPI00423463	5	2	26.84	☐	IGHG1 Putative uncharacterized protein DKFZp686O01196
0<br>0.00e+000	3<br>7.33e+007	52586.7	7.53	HUMAN	IPI00761159	5	2	26.84	☐	IGHM IGHM protein
0<br>0.00e+000	3<br>7.33e+007	52362.7	8.64	HUMAN	IPI00784817	5	2	26.84	☐	IGHV4-31 Anti-RhD monoclonal T125 gamma1 heavy chain precursor
0<br>0.00e+000	3<br>7.33e+007	52360.7	8.84	HUMAN	IPI00423464	5	2	26.84	☐	IGHG1 Putative uncharacterized protein DKFZp686K03196
0<br>0.00e+000	3<br>7.33e+007	52286.6	8.84	HUMAN	IPI00785084	5	2	26.84	☐	IGHV4-31 Immunoglobulin heavy variable 4-31
0<br>0.00e+000	3<br>7.33e+007	52121.3	7.50	HUMAN	IPI00784828	5	2	26.84	☐	- Putative uncharacterized protein DKFZp686C11235
0<br>0.00e+000	3<br>7.33e+007	52043.2	8.31	HUMAN	IPI00784842	5	2	26.84	☐	IGHV4-31 Putative uncharacterized protein DKFZp686G11190
0<br>0.00e+000	3<br>7.33e+007	51987.3	8.57	HUMAN	IPI00807531	5	2	26.84	☐	IGHG1 IGHG1 protein
0<br>0.00e+000	3<br>7.33e+007	51724.9	8.14	HUMAN	IPI00645363	5	2	26.84	☐	IGHG1 Putative uncharacterized protein DKFZp686P15220
0<br>0.00e+000	3<br>7.33e+007	51715.8	7.88	HUMAN	IPI00815926	5	2	26.84	☐	IGHG1 IGHG1 protein
0<br>0.00e+000	3<br>7.33e+007	51596.7	8.44	HUMAN	IPI00876888	5	2	26.84	☐	- cDNA FLJ78387
0<br>0.00e+000	3<br>7.33e+007	51395.4	8.69	HUMAN	IPI00448938	5	2	26.84	☐	IGHG1 IGHG1 protein
0<br>0.00e+000	3<br>7.33e+007	51344.3	7.54	HUMAN	IPI00784822	5	2	26.84	☐	IGHV4-31 IGHV4-31 protein
0<br>0.00e+000	3<br>7.33e+007	51254.2	7.88	HUMAN	IPI00829944	5	2	26.84	☐	IGHG1 IGHG1 protein
0<br>0.00e+000	3<br>7.33e+007	51204.5	8.46	HUMAN	IPI00784810	5	2	26.84	☐	IGHV4-31 IGHV4-31 protein
0<br>0.00e+000	3<br>7.33e+007	50927.0	8.33	HUMAN	IPI00816314	5	2	26.84	☐	IGHM Putative uncharacterized protein DKFZp686I15196
0<br>0.00e+000	3<br>7.33e+007	38162.4	8.27	HUMAN	IPI00816681	6	2	26.84	☐	IGHM Hepatitis B virus receptor binding protein (Fragment)
0<br>0.00e+000	2<br>5.35e+007	75553.5	6.60	HUMAN	IPI00382606	1	1	15.16	☐	F7 Factor VII active site mutant immunoconjugate
0<br>0.00e+000	2<br>5.35e+007	52420.7	7.89	HUMAN	IPI00784998	2	1	15.16	☐	- Putative uncharacterized protein DKFZp686M24218
0<br>0.00e+000	2<br>5.35e+007	51986.4	8.14	HUMAN	IPI00550640	2	1	15.16	☐	IGHG4 IGHG4 protein
0<br>0.00e+000	2<br>5.35e+007	51536.5	7.51	HUMAN	IPI00784942	2	1	15.16	☐	- Putative uncharacterized protein DKFZp686E23209
0<br>0.00e+000	2<br>5.35e+007	51325.5	6.56	HUMAN	IPI00784807	2	1	15.16	☐	IGHG2 Putative uncharacterized protein
0<br>0.00e+000	2<br>5.35e+007	51099.2	7.85	HUMAN	IPI00426051	2	1	15.16	☐	LOC100133739 Putative uncharacterized protein DKFZp686C15213
0<br>0.00e+000	2<br>5.35e+007	46061.2	7.63	HUMAN	IPI00399007	2	1	15.16	☐	IGHG2 Putative uncharacterized protein DKFZp686I04196 (Fragment)
0<br>0.00e+000	2<br>5.35e+007	43922.3	8.98	HUMAN	IPI00829767	2	1	15.16	☐	IGHG2 Uncharacterized protein IGHG2 (Fragment)

0<br>0.00e+000	2<br>5.35e+007	43372.3	8.30	HUMAN	IPI00830132	2	1	15.16	☐	IGHG4 Uncharacterized protein IGHG4 (Fragment)
0<br>0.00e+000	2<br>5.35e+007	35940.7	7.18	HUMAN	IPI00829814	3	1	15.16	☐	IGHG4 Ig gamma-4 chain C region
0<br>0.00e+000	1<br>1.13e+008	24904.2	7.79	HUMAN	IPI00844239	5	1	11.68	☐	- Immunoblobulin G1 Fab heavy chain variable region (Fragment)
0<br>0.00e+000	1<br>1.13e+008	22226.0	8.44	HUMAN	IPI00442911	6	1	11.68	☐	IGHV4-31 CDNA FLJ26266 fis, clone DMC05613
0<br>0.00e+000	9<br>1.97e+008	25038.7	5.45	HUMAN	IPI00022389	14	3	41.98	☐<br>57.1	CRP Isoform 1 of C-reactive protein precursor
0<br>0.00e+000	5<br>1.70e+008	11631.6	8.60	HUMAN	IPI00642842	9	1	9.77	☐	CRP C-reactive protein, pentraxin-related
0<br>0.00e+000	5<br>1.70e+008	10415.2	9.26	HUMAN	IPI00218876	10	1	9.77	☐	CRP Isoform 2 of C-reactive protein precursor
8<br>2.19e+008	2<br>2.88e+008	55064.7	5.97	HUMAN	IPI00029863	8	3	41.93	☐<br>58.1	SERPINF2 SERPINF2 protein
8<br>2.19e+008	2<br>2.88e+008	54566.1	5.87	HUMAN	IPI00879231	8	3	41.93	☐	SERPINF2 Alpha-2-antiplasmin precursor
8<br>2.19e+008	2<br>2.88e+008	27823.2	6.30	HUMAN	IPI00879937	16	3	41.93	☐	SERPINF2 28 kDa protein
7<br>2.31e+008	1<br>5.70e+008	18555.5	5.57	HUMAN	IPI00879608	18	2	27.64	☐	SERPINF2 19 kDa protein
3<br>8.67e+007	2<br>2.88e+008	29543.4	6.44	HUMAN	IPI00877925	10	2	26.67	☐	SERPINF2 30 kDa protein
9<br>1.37e+008	12<br>4.91e+007	25387.2	6.10	HUMAN	IPI00022391	16	3	41.23	☐<br>59.1	APCS Serum amyloid P-component precursor
17<br>4.64e+007	9<br>2.23e+008	11175.1	6.27	HUMAN	IPI00021854	31	3	39.98	☐<br>60.1	APOA2 Apolipoprotein A-II precursor
0<br>0.00e+000	7<br>1.05e+008	94973.5	5.70	HUMAN	IPI00021885	4	3	39.29	☐<br>61.1	FGA Isoform 1 of Fibrinogen alpha chain precursor
0<br>0.00e+000	7<br>1.05e+008	69757.0	8.23	HUMAN	IPI00029717	6	3	39.29	☐	FGA Isoform 2 of Fibrinogen alpha chain precursor
1<br>6.41e+006	4<br>3.69e+007	60615.1	5.63	HUMAN	IPI00479116	5	2	38.20	☐<br>62.1	CPN2 Carboxypeptidase N subunit 2 precursor
1<br>6.41e+006	4<br>3.69e+007	60585.0	5.72	HUMAN	IPI00738433	5	2	38.20	☐	CPN2 similar to Carboxypeptidase N subunit 2 precursor
5<br>5.95e+006	0<br>0.00e+000	252209.2	5.66	HUMAN	IPI00022937	2	4	38.19	☐<br>63.1	F5 Coagulation factor V
5<br>5.95e+006	0<br>0.00e+000	251672.6	5.68	HUMAN	IPI00478809	2	4	38.19	☐	F5 Coagulation factor V precursor
0<br>0.00e+000	4<br>8.78e+007	68611.0	6.86	HUMAN	IPI00479708	3	2	38.03	☐<br>64.1	IGHM IGHM protein
0<br>0.00e+000	4<br>8.78e+007	68125.5	6.44	HUMAN	IPI00884180	3	2	38.03	☐	IGHM IGHM protein
0<br>0.00e+000	4<br>8.78e+007	67296.3	5.89	HUMAN	IPI00477090	3	2	38.03	☐	IGHM IGHM protein
0<br>0.00e+000	4<br>8.78e+007	66184.9	6.53	HUMAN	IPI00549291	3	2	38.03	☐	IGHM IGHM protein
0<br>0.00e+000	4<br>8.78e+007	65305.2	8.10	HUMAN	IPI00884141	3	2	38.03	☐	IGHM IGHM protein
0<br>0.00e+000	4<br>8.78e+007	65301.2	8.10	HUMAN	IPI00884293	3	2	38.03	☐	IGHM IGHM protein
0<br>0.00e+000	4<br>8.78e+007	65291.2	8.45	HUMAN	IPI00884452	3	2	38.03	☐	IGHM IGHM protein
0<br>0.00e+000	4<br>8.78e+007	65275.1	8.10	HUMAN	IPI00883614	3	2	38.03	☐	IGHM IGHM protein

0<br>0.00e+000	4<br>8.78e+007	65039.5	6.34	HUMAN	IPI00828205	3	2	38.03	☐	IGHM IGHM protein
0<br>0.00e+000	3<br>8.89e+007	43057.5	5.12	HUMAN	IPI00385264	2	1	19.62	☐	- Ig mu heavy chain disease protein
1<br>9.04e+006	5<br>9.30e+007	55928.5	8.54	HUMAN	IPI00298497	5	2	36.56	☐<br>65.1	FGB Fibrinogen beta chain precursor
3<br>1.02e+007	4<br>5.09e+007	12815.6	7.90	HUMAN	IPI00657670	23	2	35.90	☐<br>66.1	- Apolipoprotein C-III variant 1
3<br>1.02e+007	4<br>5.09e+007	10852.4	5.23	HUMAN	IPI00021857	27	2	35.90	☐	APOC3 Apolipoprotein C-III precursor
1<br>7.20e+006	7<br>1.14e+007	431766.7	9.22	HUMAN	IPI00009286	1	4	35.74	☐<br>67.1	MLL Isoform 1 of Zinc finger protein HRX
1<br>7.20e+006	7<br>1.14e+007	427735.0	9.27	HUMAN	IPI00218500	1	4	35.74	☐	MLL Isoform 14P-18B of Zinc finger protein HRX
1<br>7.20e+006	1<br>3.33e+006	54317.3	11.14	HUMAN	IPI00155551	1	1	12.13	☐	MLL MLL (Fragment)
2<br>2.20e+007	0<br>0.00e+000	28357.7	5.05	HUMAN	IPI00025862	10	2	34.47	☐<br>68.1	C4BPB Isoform 1 of C4b-binding protein beta chain precursor
2<br>2.20e+007	0<br>0.00e+000	28286.6	5.05	HUMAN	IPI00555752	10	2	34.47	☐	C4BPB Isoform 2 of C4b-binding protein beta chain precursor
1<br>1.29e+007	0<br>0.00e+000	20298.1	5.03	HUMAN	IPI00643437	6	1	15.14	☐	C4BPB Complement component 4 binding protein, beta
11<br>2.97e+007	0<br>0.00e+000	46324.8	5.87	HUMAN	IPI00292946	4	2	33.22	☐<br>69.1	SERPINA7 Thyroxine-binding globulin precursor
7<br>3.12e+007	2<br>1.09e+008	32117.4	6.60	HUMAN	IPI00807459	11	2	32.83	☐<br>70.1	IGKC IGKC protein
7<br>3.12e+007	2<br>1.09e+008	26245.8	8.57	HUMAN	IPI00440577	13	2	32.83	☐	IGKV2-24 IGKV2-24 protein
7<br>3.12e+007	2<br>1.09e+008	26234.7	8.24	HUMAN	IPI00550731	13	2	32.83	☐	- Putative uncharacterized protein
7<br>3.12e+007	2<br>1.09e+008	26234.6	6.30	HUMAN	IPI00419424	13	2	32.83	☐	IGKV1-5 IGKV1-5 protein
7<br>3.12e+007	2<br>1.09e+008	26024.4	5.93	HUMAN	IPI00854806	13	2	32.83	☐	IGKV1-5 IGKV1-5 protein
7<br>3.12e+007	2<br>1.09e+008	25936.4	8.69	HUMAN	IPI00472961	13	2	32.83	☐	IGKC IGKC protein
7<br>3.12e+007	2<br>1.09e+008	25924.3	8.43	HUMAN	IPI00816118	13	2	32.83	☐	IGKC IGKC protein
7<br>3.12e+007	2<br>1.09e+008	25871.2	6.72	HUMAN	IPI00478600	13	2	32.83	☐	IGKV1-5 IGKV1-5 protein
7<br>3.12e+007	2<br>1.09e+008	25834.0	6.14	HUMAN	IPI00889156	13	2	32.83	☐	IGKV3-20 IGK@ protein
7<br>3.12e+007	2<br>1.09e+008	25807.2	8.17	HUMAN	IPI00784661	13	2	32.83	☐	- Putative uncharacterized protein
7<br>3.12e+007	2<br>1.09e+008	25773.0	5.94	HUMAN	IPI00784865	13	2	32.83	☐	IGK@ IGK@ protein
7<br>3.12e+007	2<br>1.09e+008	25765.0	5.75	HUMAN	IPI00430820	14	2	32.83	☐	IGKV1-5 IGKV1-5 protein
7<br>3.12e+007	2<br>1.09e+008	25751.1	7.54	HUMAN	IPI00827488	13	2	32.83	☐	IGKC IGKC protein
7<br>3.12e+007	2<br>1.09e+008	25741.2	8.49	HUMAN	IPI00784070	13	2	32.83	☐	IGKC IGKC protein
7<br>3.12e+007	2<br>1.09e+008	25707.1	8.17	HUMAN	IPI00430847	13	2	32.83	☐	IGKC IGKC protein
7<br>3.12e+007	2<br>1.09e+008	25702.1	7.51	HUMAN	IPI00784773	13	2	32.83	☐	- Putative uncharacterized protein

7<br>3.12e+007	2<br>1.09e+008	25697.9	8.69	HUMAN	IPI00853045	14	2	32.83	☐	IGKC Anti-RhD monoclonal T125 kappa light chain precursor
7<br>3.12e+007	2<br>1.09e+008	25674.1	8.62	HUMAN	IPI00761125	14	2	32.83	☐	IGKC IGKC protein
7<br>3.12e+007	2<br>1.09e+008	25602.9	7.55	HUMAN	IPI00746963	13	2	32.83	☐	IGKC IGKC protein
7<br>3.12e+007	2<br>1.09e+008	25520.8	6.14	HUMAN	IPI00784985	14	2	32.83	☐	IGK@ IGK@ protein
7<br>3.12e+007	2<br>1.09e+008	25389.7	6.30	HUMAN	IPI00845354	14	2	32.83	☐	IGKC IGKC protein
7<br>3.12e+007	2<br>1.09e+008	24030.1	8.29	HUMAN	IPI00430808	15	2	32.83	☐	IGKC Immunoblobulin light chain (Fragment)
6<br>2.82e+007	0<br>0.00e+000	20668.5	5.13	HUMAN	IPI00556287	8	1	13.31	☐	- Putative uncharacterized protein
3<br>9.43e+007	0<br>0.00e+000	83268.3	7.23	HUMAN	IPI00303963	4	3	32.45	☐<br>71.1	C2 Complement C2 precursor (Fragment)
3<br>9.43e+007	0<br>0.00e+000	58789.4	7.88	HUMAN	IPI00643506	7	3	32.45	☐	C2 Complement component 2
1<br>2.03e+008	0<br>0.00e+000	39350.0	5.89	HUMAN	IPI00645500	2	1	12.18	☐	C2 Complement component 2
1<br>1.29e+008	2<br>1.02e+008	38429.2	6.16	HUMAN	IPI00020986	5	2	32.21	☐<br>72.1	LUM Lumican precursor
1<br>1.29e+008	2<br>1.02e+008	23151.8	8.52	HUMAN	IPI00794403	9	2	32.21	☐	LUM 23 kDa protein
1<br>1.29e+008	1<br>9.96e+007	26325.4	6.65	HUMAN	IPI00796888	4	1	16.24	☐	LUM 26 kDa protein
0<br>0.00e+000	3<br>1.15e+008	46342.5	5.97	HUMAN	IPI00006114	5	2	31.95	☐<br>73.1	SERPINF1 Pigment epithelium-derived factor precursor
0<br>0.00e+000	2<br>3.07e+007	24515.3	5.84	HUMAN	IPI00796279	4	1	17.86	☐	SERPINF1 25 kDa protein
0<br>0.00e+000	1<br>2.83e+008	12025.8	4.72	HUMAN	IPI00790473	12	1	14.09	☐	SERPINF1 12 kDa protein
10<br>1.09e+008	0<br>0.00e+000	68000.7	7.62	HUMAN	IPI00394992	4	2	31.35	☐<br>74.1	PGLYRP2 Isoform 2 of N-acetylmuramoyl-L-alanine amidase precursor
10<br>1.09e+008	0<br>0.00e+000	62217.3	7.25	HUMAN	IPI00163207	4	2	31.35	☐	PGLYRP2 Isoform 1 of N-acetylmuramoyl-L-alanine amidase precursor
3<br>2.68e+007	0<br>0.00e+000	158538.0	7.24	HUMAN	IPI00027235	2	3	30.07	☐<br>75.1	ATRN Isoform 1 of Attractin precursor
3<br>2.68e+007	0<br>0.00e+000	150810.8	7.14	HUMAN	IPI00218460	2	3	30.07	☐	ATRN Isoform 3 of Attractin precursor
3<br>2.68e+007	0<br>0.00e+000	141429.8	6.65	HUMAN	IPI00162735	2	3	30.07	☐	ATRN Isoform 2 of Attractin precursor
4<br>6.36e+007	0<br>0.00e+000	46723.3	6.28	HUMAN	IPI00641737	6	2	29.00	☐<br>76.1	HP Haptoglobin precursor
4<br>6.36e+007	0<br>0.00e+000	43055.8	6.45	HUMAN	IPI00607707	6	2	29.00	☐	HPR Isoform 2 of Haptoglobin-related protein precursor
4<br>6.36e+007	0<br>0.00e+000	39007.7	6.41	HUMAN	IPI00477597	7	2	29.00	☐	HPR Isoform 1 of Haptoglobin-related protein precursor
4<br>6.36e+007	0<br>0.00e+000	38452.0	6.13	HUMAN	IPI00478493	7	2	29.00	☐	HP HP protein
1<br>1.72e+006	0<br>0.00e+000	31382.1	8.48	HUMAN	IPI00431645	3	1	9.90	☐	HP HP protein
2<br>6.14e+006	2<br>8.17e+007	92336.9	5.91	HUMAN	IPI00299503	3	2	28.91	☐<br>77.1	GPLD1 Isoform 1 of Phosphatidylinositol-glycan-specific phospholipase D precursor
0<br>0.00e+000	3<br>8.72e+007	14806.8	9.27	HUMAN	IPI00019399	14	2	26.15	☐<br>78.1	SAA4 Serum amyloid A-4 protein precursor

2<br>1.28e+008	0<br>0.00e+000	25505.6	8.20	HUMAN	IPI00026199	12	2	24.96	☐<br>79.1	GPX3 Glutathione peroxidase 3 precursor
2<br>4.89e+008	0<br>0.00e+000	65163.6	6.07	HUMAN	IPI00011252	4	2	24.93	☐<br>80.1	C8A Complement component C8 alpha chain precursor
1<br>4.44e+006	2<br>5.49e+008	45141.1	5.64	HUMAN	IPI00027482	6	2	24.61	☐<br>81.1	SERPINA6 Corticosteroid-binding globulin precursor
0<br>0.00e+000	2<br>3.76e+007	27745.3	4.73	HUMAN	IPI00021263	8	2	24.12	☐<br>82.1	YWHAZ 14-3-3 protein zeta/delta
0<br>0.00e+000	2<br>3.76e+007	27742.2	4.65	HUMAN	IPI00879359	8	2	24.12	☐	- 28 kDa protein
0<br>0.00e+000	1<br>5.49e+007	19072.5	4.48	HUMAN	IPI00789337	7	1	13.16	☐	YWHAZ cDNA, FLJ79516, highly similar to 14-3-3 protein zeta/delta
0<br>0.00e+000	2<br>7.40e+007	10931.7	8.89	HUMAN	IPI00220362	13	1	23.59	☐<br>83.1	HSPE1 10 kDa heat shock protein, mitochondrial
0<br>0.00e+000	4<br>1.67e+007	57937.2	7.95	HUMAN	IPI00479186	4	2	23.09	☐<br>84.1	PKM2 Isoform M2 of Pyruvate kinase isozymes M1/M2
0<br>0.00e+000	3<br>2.15e+007	58062.4	7.61	HUMAN	IPI00220644	2	1	15.32	☐	PKM2 Isoform M1 of Pyruvate kinase isozymes M1/M2
0<br>0.00e+000	3<br>2.15e+007	39592.8	6.51	HUMAN	IPI00888126	3	1	15.32	☐	LOC652797 similar to pyruvate kinase, muscle
0<br>0.00e+000	3<br>2.15e+007	26586.6	5.42	HUMAN	IPI00607698	5	1	15.32	☐	PKM2 27 kDa protein
0<br>0.00e+000	3<br>2.15e+007	26383.3	5.42	HUMAN	IPI00788663	5	1	15.32	☐	PKM2 26 kDa protein
0<br>0.00e+000	3<br>2.15e+007	24333.7	6.50	HUMAN	IPI00792817	5	1	15.32	☐	PKM2 24 kDa protein
0<br>0.00e+000	3<br>2.15e+007	22725.2	6.31	HUMAN	IPI00789727	6	1	15.32	☐	PKM2 23 kDa protein
0<br>0.00e+000	3<br>2.15e+007	18869.9	9.47	HUMAN	IPI00797668	7	1	15.32	☐	PKM2 19 kDa protein
0<br>0.00e+000	3<br>2.15e+007	10094.7	6.49	HUMAN	IPI00798295	14	1	15.32	☐	PKM2 10 kDa protein
0<br>0.00e+000	1<br>2.21e+006	37276.3	8.47	HUMAN	IPI00847989	3	1	7.77	☐	PKM2 Pyruvate kinase (Fragment)
3<br>5.37e+007	0<br>0.00e+000	52286.5	6.86	HUMAN	IPI00010295	6	2	22.96	☐<br>85.1	CPN1 Carboxypeptidase N catalytic chain precursor
2<br>7.94e+007	0<br>0.00e+000	22879.6	5.17	HUMAN	IPI00641144	7	1	13.10	☐	CPN1 Carboxypeptidase N, polypeptide 1
1<br>4.84e+006	1<br>3.52e+007	24390.7	6.90	HUMAN	IPI00514285	7	1	21.85	☐<br>86.1	PTGDS Prostaglandin D2 synthase 21kDa
1<br>4.84e+006	1<br>3.52e+007	22949.1	9.92	HUMAN	IPI00513767	7	1	21.85	☐	PTGDS Prostaglandin D2 synthase 21kDa
1<br>4.84e+006	1<br>3.52e+007	21028.9	7.65	HUMAN	IPI00013179	8	1	21.85	☐	PTGDS Prostaglandin-H2 D-isomerase precursor
1<br>4.84e+006	1<br>3.52e+007	16871.3	9.45	HUMAN	IPI00514208	10	1	21.85	☐	PTGDS Prostaglandin D2 synthase 21kDa
2<br>1.26e+009	0<br>0.00e+000	39749.5	5.08	HUMAN	IPI00218732	4	1	20.06	☐<br>87.1	PON1 Serum paraoxonase/arylesterase 1
2<br>1.26e+009	0<br>0.00e+000	31865.4	4.82	HUMAN	IPI00798167	5	1	20.06	☐	PON1 32 kDa protein
1<br>2.88e+007	1<br>5.16e+006	67047.3	8.50	HUMAN	IPI00294395	2	1	18.94	☐<br>88.1	C8B Complement component C8 beta chain precursor
0<br>0.00e+000	1<br>1.12e+008	51778.8	5.34	HUMAN	IPI00296176	1	1	18.12	☐<br>89.1	F9 Coagulation factor IX precursor

1 8.37e+006	0 0.00e+000	43618.1	6.96	HUMAN	IPI00218795	2	1	17.87	90.1	SELL L-selectin precursor
0 0.00e+000	2 2.01e+008	28870.3	6.59	HUMAN	IPI00215983	4	1	17.82	91.1	CA1 Carbonic anhydrase 1
0 0.00e+000	2 2.01e+008	21549.3	7.10	HUMAN	IPI00796435	6	1	17.82		CA1 22 kDa protein
0 0.00e+000	2 2.01e+008	16254.8	9.48	HUMAN	IPI00788926	8	1	17.82		CA1 16 kDa protein
0 0.00e+000	2 2.01e+008	14008.1	9.21	HUMAN	IPI00798267	9	1	17.82		CA1 14 kDa protein
2 2.77e+007	0 0.00e+000	83268.7	5.69	HUMAN	IPI00884176	1	1	16.71	92.1	F13A1 coagulation factor XIII A1 subunit precursor
2 2.77e+007	0 0.00e+000	83267.7	5.75	HUMAN	IPI00297550	1	1	16.71		F13A1 Coagulation factor XIII A chain precursor
1 9.94e+006	0 0.00e+000	51276.8	8.32	HUMAN	IPI00021364	4	1	16.62	93.1	CFP Properdin precursor
2 6.26e+007	0 0.00e+000	70682.3	6.99	HUMAN	IPI00029193	2	1	16.10	94.1	HGFAC Hepatocyte growth factor activator precursor
0 0.00e+000	1 4.32e+006	91614.9	5.85	HUMAN	IPI00871139	1	1	15.77	95.1	MASP1 92 kDa protein
0 0.00e+000	1 4.32e+006	79259.3	5.41	HUMAN	IPI00299307	1	1	15.77		MASP1 Complement-activating component of Ra-reactive factor precursor
1 9.91e+006	0 0.00e+000	54641.0	4.95	HUMAN	IPI00029658	2	1	15.73	96.1	EFEMP1 Isoform 1 of EGF-containing fibulin-like extracellular matrix protein 1 precursor
1 9.91e+006	0 0.00e+000	54583.9	4.95	HUMAN	IPI00220814	2	1	15.73		EFEMP1 Isoform 3 of EGF-containing fibulin-like extracellular matrix protein 1 precursor
1 9.91e+006	0 0.00e+000	54553.9	4.95	HUMAN	IPI00220815	2	1	15.73		EFEMP1 Isoform 4 of EGF-containing fibulin-like extracellular matrix protein 1 precursor
1 9.91e+006	0 0.00e+000	53722.8	4.90	HUMAN	IPI00220813	2	1	15.73		EFEMP1 Isoform 2 of EGF-containing fibulin-like extracellular matrix protein 1 precursor
0 0.00e+000	1 1.04e+008	54732.0	5.68	HUMAN	IPI00019576	2	1	15.67	97.1	F10 Coagulation factor X precursor
0 0.00e+000	1 1.04e+008	37094.7	5.63	HUMAN	IPI00552633	3	1	15.67		F10 Coagulation factor X
0 0.00e+000	1 8.41e+006	45199.8	8.98	HUMAN	IPI00030363	4	1	15.67	98.1	ACAT1 Acetyl-CoA acetyltransferase, mitochondrial precursor
0 0.00e+000	1 8.41e+006	17201.5	9.98	HUMAN	IPI00062003	11	1	15.67		ACAT1 ACAT1 protein
1 1.72e+007	0 0.00e+000	42155.7	8.71	HUMAN	IPI00829918	5	1	15.57	99.1	- NPAL2 protein
0 0.00e+000	1 8.08e+007	72698.0	6.49	HUMAN	IPI00031425	2	1	15.55	100.1	HAL Histidine ammonia-lyase
2 4.88e+008	0 0.00e+000	50704.6	6.62	HUMAN	IPI00013212	2	1	15.52	101.1	CSK Tyrosine-protein kinase CSK
7 1.94e+008	2 8.86e+008	23010.1	5.76	HUMAN	IPI00022420	4	1	15.46	102.1	RBP4 Plasma retinol-binding protein precursor
7 1.94e+008	2 8.86e+008	22973.9	5.77	HUMAN	IPI00844536	5	1	15.46		RBP4 Uncharacterized protein RBP4
7 1.94e+008	2 8.86e+008	22943.9	5.77	HUMAN	IPI00480192	5	1	15.46		RBP4 Retinol binding protein 4, plasma
0 0.00e+000	2 7.70e+006	40076.4	5.84	HUMAN	IPI00029260	4	1	15.34	103.1	CD14 Monocyte differentiation antigen CD14 precursor
3 2.90e+007	0 0.00e+000	43779.4	6.22	HUMAN	IPI00023019	3	1	15.08	104.1	SHBG Isoform 1 of Sex hormone-binding globulin precursor

3<br>2.90e+007	0<br>0.00e+000	31829.5	5.93	HUMAN	IPI00219583	4	1	15.08	☐	SHBG Isoform 2 of Sex hormone-binding globulin precursor
3<br>2.90e+007	0<br>0.00e+000	28666.3	5.32	HUMAN	IPI00884913	5	1	15.08	☐	- Sex hormone binding globulin (Fragment)
1<br>1.78e+007	0<br>0.00e+000	26721.9	8.83	HUMAN	IPI00477992	3	1	14.87	☐<br>105.1	C1QB complement component 1, q subcomponent, B chain precursor
1<br>1.78e+007	0<br>0.00e+000	24380.2	9.26	HUMAN	IPI00643948	3	1	14.87	☐	C1QB Complement component 1, q subcomponent, B chain
0<br>0.00e+000	1<br>7.68e+006	35531.5	8.92	HUMAN	IPI00291006	3	1	14.63	☐<br>106.1	MDH2 Malate dehydrogenase, mitochondrial precursor
0<br>0.00e+000	1<br>1.48e+007	47716.4	5.92	HUMAN	IPI00012007	2	1	13.86	☐<br>107.1	AHCY Adenosylhomocysteinase
2<br>7.12e+006	1<br>7.90e+006	26413.8	5.43	HUMAN	IPI00020019	6	1	13.86	☐<br>108.1	ADIPOQ Adiponectin precursor
0<br>0.00e+000	1<br>1.42e+008	10834.6	6.51	HUMAN	IPI00007047	11	1	13.72	☐<br>109.1	S100A8 Protein S100-A8
0<br>0.00e+000	1<br>2.33e+006	76684.9	4.86	HUMAN	IPI00017696	1	1	13.66	☐<br>110.1	C1S Complement C1s subcomponent precursor
0<br>0.00e+000	1<br>2.33e+006	75906.0	5.09	HUMAN	IPI00749179	1	1	13.66	☐	C1S Uncharacterized protein C1S
0<br>0.00e+000	1<br>2.33e+006	20585.3	5.00	HUMAN	IPI00790679	4	1	13.66	☐	C1S 21 kDa protein
1<br>4.59e+007	0<br>0.00e+000	78182.0	9.32	HUMAN	IPI00398310	1	1	13.65	☐<br>111.1	ZNF573 Isoform 1 of Zinc finger protein 573
1<br>4.59e+007	0<br>0.00e+000	70843.5	9.40	HUMAN	IPI00418297	1	1	13.65	☐	ZNF573 Isoform 3 of Zinc finger protein 573
1<br>4.59e+007	0<br>0.00e+000	67866.2	9.42	HUMAN	IPI00827652	1	1	13.65	☐	ZNF573 Isoform 4 of Zinc finger protein 573
1<br>4.59e+007	0<br>0.00e+000	67782.0	9.41	HUMAN	IPI00827764	1	1	13.65	☐	ZNF573 Isoform 2 of Zinc finger protein 573
1<br>1.59e+007	1<br>1.51e+007	74681.3	7.62	HUMAN	IPI00018219	1	1	13.04	☐<br>112.1	TGFBI Transforming growth factor-beta-induced protein ig-h3 precursor
1<br>1.59e+007	1<br>1.51e+007	45414.4	6.24	HUMAN	IPI00873923	2	1	13.04	☐	TGFBI TGFBI protein
1<br>1.59e+007	1<br>1.51e+007	25846.7	5.33	HUMAN	IPI00556324	4	1	13.04	☐	TGFBI Transforming growth factor, beta-induced, 68kDa variant (Fragment)
0<br>0.00e+000	1<br>1.60e+007	55114.1	7.22	HUMAN	IPI00007199	3	1	12.97	☐<br>113.1	SERPINA10 Protein Z-dependent protease inhibitor precursor
0<br>0.00e+000	1<br>1.40e+008	25977.2	6.29	HUMAN	IPI00382938	6	1	12.75	☐<br>114.1	IGLV4-3 IGLV4-3 protein
0<br>0.00e+000	1<br>1.40e+008	25148.3	6.19	HUMAN	IPI00719373	6	1	12.75	☐	IGL@ IGL@ protein
0<br>0.00e+000	1<br>1.40e+008	25108.2	8.17	HUMAN	IPI00784711	6	1	12.75	☐	- Putative uncharacterized protein
0<br>0.00e+000	1<br>1.40e+008	25024.1	7.59	HUMAN	IPI00785200	6	1	12.75	☐	- Putative uncharacterized protein
0<br>0.00e+000	1<br>1.40e+008	25021.1	8.14	HUMAN	IPI00658130	6	1	12.75	☐	IGL@ IGL@ protein
0<br>0.00e+000	1<br>1.40e+008	25015.1	5.94	HUMAN	IPI00784519	6	1	12.75	☐	- Putative uncharacterized protein
0<br>0.00e+000	1<br>1.40e+008	25002.3	6.88	HUMAN	IPI00784589	6	1	12.75	☐	- Putative uncharacterized protein DKFZp781M0386
0<br>0.00e+000	1<br>1.40e+008	24969.9	6.51	HUMAN	IPI00785079	6	1	12.75	☐	- Putative uncharacterized protein
0<br>0.00e+000	1<br>1.40e+008	24960.9	5.21	HUMAN	IPI00829626	6	1	12.75	☐	IGL@ IGL@ protein

0<br>0.00e+000	1<br>1.40e+008	24950.0	6.41	HUMAN	IPI00784983	6	1	12.75	☐	- Putative uncharacterized protein
0<br>0.00e+000	1<br>1.40e+008	24927.9	6.81	HUMAN	IPI00784713	6	1	12.75	☐	- Putative uncharacterized protein
0<br>0.00e+000	1<br>1.40e+008	24909.9	6.19	HUMAN	IPI00785050	6	1	12.75	☐	- Putative uncharacterized protein
0<br>0.00e+000	1<br>1.40e+008	24898.9	6.50	HUMAN	IPI00815938	6	1	12.75	☐	IGLV3-21 IGLV3-21 protein
0<br>0.00e+000	1<br>1.40e+008	24887.9	6.28	HUMAN	IPI00450309	6	1	12.75	☐	IGL@ IGL@ protein
0<br>0.00e+000	1<br>1.40e+008	24866.9	5.23	HUMAN	IPI00550162	6	1	12.75	☐	IGLV3-25 IGLV3-25 protein
0<br>0.00e+000	1<br>1.40e+008	24857.8	7.59	HUMAN	IPI00745660	6	1	12.75	☐	IGL@ IGL@ protein
0<br>0.00e+000	1<br>1.40e+008	24854.9	6.29	HUMAN	IPI00829640	6	1	12.75	☐	IGL@ IGL@ protein
0<br>0.00e+000	1<br>1.40e+008	24852.9	8.74	HUMAN	IPI00885076	6	1	12.75	☐	IGLC2;IGLV2-14;IGLC3;IGLC1 IGLV2-14 protein
0<br>0.00e+000	1<br>1.40e+008	24823.7	6.28	HUMAN	IPI00744476	6	1	12.75	☐	IGL@ IGL@ protein
0<br>0.00e+000	1<br>1.40e+008	24803.7	7.60	HUMAN	IPI00784627	6	1	12.75	☐	- Putative uncharacterized protein
0<br>0.00e+000	1<br>1.40e+008	24802.0	5.92	HUMAN	IPI00888118	6	1	12.75	☐	IGLC1 IGLC1 protein
0<br>0.00e+000	1<br>1.40e+008	24799.7	6.88	HUMAN	IPI00555945	6	1	12.75	☐	IGL@ IGL@ protein
0<br>0.00e+000	1<br>1.40e+008	24792.7	5.93	HUMAN	IPI00154742	6	1	12.75	☐	IGL@ IGL@ protein
0<br>0.00e+000	1<br>1.40e+008	24746.9	5.66	HUMAN	IPI00807428	6	1	12.75	☐	- Putative uncharacterized protein
0<br>0.00e+000	1<br>1.40e+008	24712.7	6.88	HUMAN	IPI00785164	6	1	12.75	☐	- Putative uncharacterized protein
0<br>0.00e+000	1<br>1.40e+008	24700.6	6.40	HUMAN	IPI00719452	6	1	12.75	☐	IGL@ IGL@ protein
0<br>0.00e+000	1<br>1.40e+008	24696.8	5.89	HUMAN	IPI00718819	6	1	12.75	☐	IGL@;IGLC2;IGLV2-14;IGLC3;IGLC1 IGLV2-14 protein
0<br>0.00e+000	1<br>1.40e+008	24654.7	7.54	HUMAN	IPI00827875	6	1	12.75	☐	- Lambda-chain precursor
0<br>0.00e+000	1<br>1.40e+008	24653.8	5.94	HUMAN	IPI00784935	6	1	12.75	☐	IGL@ IGL@ protein
0<br>0.00e+000	1<br>1.40e+008	24632.6	6.88	HUMAN	IPI00785196	6	1	12.75	☐	- Putative uncharacterized protein
0<br>0.00e+000	1<br>1.40e+008	24509.3	6.81	HUMAN	IPI00816555	6	1	12.75	☐	IGLV2-14 IGLV2-14 protein
0<br>0.00e+000	1<br>1.40e+008	11403.8	7.89	HUMAN	IPI00852577	14	1	12.75	☐	- C1 segment protein
0<br>0.00e+000	1<br>1.40e+008	11358.8	8.49	HUMAN	IPI00642632	14	1	12.75	☐	- C7 protein
0<br>0.00e+000	1<br>1.40e+008	11302.7	6.91	HUMAN	IPI00830047	14	1	12.75	☐	- Uncharacterized protein ENSP00000374858 (Fragment)
3<br>2.21e+008	0<br>0.00e+000	135578.1	5.13	HUMAN	IPI00300371	0	1	12.32	☐<br>115.1	SF3B3 Isoform 1 of Splicing factor 3B subunit 3
3<br>2.21e+008	0<br>0.00e+000	30209.9	6.07	HUMAN	IPI00179138	4	1	12.32	☐	SF3B3 Isoform 2 of Splicing factor 3B subunit 3
1<br>3.45e+007	0<br>0.00e+000	72883.7	7.48	HUMAN	IPI00025864	1	1	11.93	☐<br>116.1	BCHE Cholinesterase precursor

0<br>0.00e+000	1<br>4.24e+006	25322.6	8.15	HUMAN	IPI00029235	5	1	11.81	☐<br>117.1	IGFBP6 Insulin-like growth factor-binding protein 6 precursor
0<br>0.00e+000	1<br>5.27e+007	99849.7	5.49	HUMAN	IPI00028413	1	1	11.77	☐<br>118.1	ITIH3 Isoform 1 of Inter-alpha-trypsin inhibitor heavy chain H3 precursor
0<br>0.00e+000	1<br>5.27e+007	99328.1	5.52	HUMAN	IPI00876950	1	1	11.77	☐	ITIH3 Isoform 2 of Inter-alpha-trypsin inhibitor heavy chain H3 precursor
0<br>0.00e+000	1<br>5.27e+007	75078.5	5.59	HUMAN	IPI00873416	2	1	11.77	☐	ITIH3 Uncharacterized protein ITIH3
0<br>0.00e+000	1<br>7.89e+006	78439.4	6.88	HUMAN	IPI00015481	1	1	11.02	☐<br>119.1	ZNF408 Zinc finger protein 408

Summarize Results for Review

Summarize | Save Settings | Reset

Mode: Protein - Protein Comparison Redundant

Group results by: ○ File ◉ Directory

Data directories: Select ...

☑ 101215TMMU_HJY
☑ 101220TMMU_HJY

Search result files:

*.spo

Validation and Sorting

Filter results by: valid

Protein grouping method: 1 shared peptide

Sort proteins by: Score

Filter by protein score: > 0

Filter peptides by:

Score: > 0 **% SPI:** > 0

Required AAs: any **Disallowed AAs:** none

Accession #'s:

Review Fields

☑ Filename
☑ Score
☑ Mean Intensity
☑ Protein MW
☑ Protein pI
☑ Species
☑ Accession #
☑ Protein name
☐ Excel export
☐ DEQ ratios ☐ Invert
☐ iTRAQ ratios control: 114
☐ Category
