# Supplementary material for: Identification of Differentially Expressed Serum Proteins in Infectious Purpura Fulminans
Source: Dis Markers. 2014 Feb 10;2014:698383. doi: 10.1155/2014/698383 (PMC3934775; doi:10.1155/2014/698383)
Supplement: Supplementary file 1 — S1: The raw result of the Spectrum Mill proteomics analysis. Supplemental materials: The MS parameters and the database search parameters setting. Table S1: The raw result of biological processes based on GO analysis by DAVID. Table S2: The raw result of molecular functions based on GO analysis by DAVID. Table S3: The raw result of cellular components based on GO analysis by DAVID. Table S4: The raw result of physiological pathways enrichment analysis by DAVID. [file 698383.f1.zip › supplementary materials.doc]

**HPLC-Chip/MS analysis**

Data-dependent MS acquisition was performed on theAgilent LC/MSD Trap XCT with the following MS conditions: 4 L/min, 300°C; skim 1: 30 V; capillary exit: 75 V; capillary voltage: 1800 V; for each precursor ion two averages were taken; ion current control: on; trap drive: 85; smart target: 500,000; MS scan range: 400–1600; maximum accumulation time: 150 ms; ultra scan: on averages: 1; fragmentation amplitude: 1.25 V; MS/MS: number of parents: 5; SmartFrag: on, 30–200%; spectra were actively excluded for fragmentation after two recorded spectra for 1 min to allow the detection of less abundant co-eluting compounds; exclude +1: on, MS/MS scan range: 100–2000; prefer +2: on; ion current control target: 500,000; ultra scan: on.

**Database search**

Peak lists were created with the Spectrum Mill Data Extractor program under the following conditions: scanswith the same precursor ±1.4 m/z were merged within a time frame of ±15 s. Charges up to a maximum of 5 were assigned to the precursor ion, and the ^12^C peak was determined by the Data Extractor. Precursor ions needed to have a minimum signal to noise value of 25. Two missed cleavages were allowed. Peptides were automatically identified by the Spectrum Mill software using IPI human database (version 3.43) for tryptic peptides with the restriction to Homo sapiens. A mass tolerance of ±2.5Da for the precursor ions and a tolerance of ±0.7Da for the fragment ions were used. A Spectrum Mill autovalidation was performed first in the protein details mode and then in the peptide mode. Minimum scores, minimum scored peak intensity (SPI), forward minus reversed score threshold, and rank 1 minus rank 2 score threshold for peptides were dependent on the assigned precursor charge.

**Table S1 Database search settings**

| Database: NCBI rattus norvegicus database (5/4/2008) | | | | |
| --- | --- | --- | --- | --- |
| Protein details search mode (Filter proteins by score: >25) | | | | |
| Precursor Charge | Score Threshold | %SPI Threshold | Fwd - Rev Score Threshold | Rank1-2 score Threshold |
| 2 | 6 | 70% | 2 | 2 |
| 2 | 6 | 90% | 1 | 1 |
| 3 | 8 | 70% | 2 | 2 |
| 4 | 8 | 70% | 2 | 2 |
| 5 | 12 | 70% | 2 | 2 |
| Peptide search mode | | | | |
| Precursor Charge | Score Threshold | %SPI Threshold | Fwd - Rev Score Threshold | Rank1-2 score Threshold |
| 2 | 11 | 70% | 2 | 2 |
| 3 | 13 | 70% | 2 | 2 |
| 4 | 13 | 70% | 2 | 2 |
| 5 | 15 | 70% | 2 | 2 |
